# Sex as a Determinant of Responses to a Coronary Artery Disease Self-Antigen Identified by Immune-Peptidomics

**DOI:** 10.3389/fimmu.2020.00694

**Published:** 2020-04-21

**Authors:** Wai Man Lio, Bojan Cercek, Juliana Yano, Wei Yang, Jonathan Ghermezi, Xiaoning Zhao, Jianchang Zhou, Bo Zhou, Michael R. Freeman, Kuang-Yuh Chyu, Prediman K. Shah, Paul C. Dimayuga

**Affiliations:** ^1^Oppenheimer Atherosclerosis Research Center, Department of Cardiology, Smidt Heart Institute, Cedars-Sinai Medical Center, Los Angeles, CA, United States; ^2^Division of Cancer Biology and Therapeutics, Departments of Surgery and Biomedical Sciences, Cedars-Sinai Medical Center, Los Angeles, CA, United States

**Keywords:** coronary artery disease, atherosclerosis, immune-peptidome, self-antigen, sexual dimorphism

## Abstract

A significant body of work implicates the adaptive immune response in atherosclerosis, the main underlying cause of coronary artery disease (CAD), yet specific antigens involved remain to be fully identified. The pathobiology of CAD is influenced by sex with many factors that may be involved in the underlying mechanisms. Given the reported sexual dimorphic nature of immune-inflammatory responses, we investigated the influence of sex on potential CAD self-antigens from acute coronary syndrome (ACS) patients using immune-precipitation of soluble HLA Class-I/peptide complexes and mass spectrometry. Relevance of identified self-antigens to atherosclerosis, the major underlying cause of CAD, was tested in the apoE–/– atherosclerotic mouse model. Soluble HLA Class-I complexes from ACS patients and self-reported controls were immune-precipitated and subjected to elution, denaturation and size-exclusion to obtain HLA-bound peptides. Peptides were then subjected to mass spectrometry and patient-unique self-peptides were grouped as common to both female and male, or unique to either sex. Three peptides common to both female and male patients (COL6A1, CDSN, and SAA2), and 2 peptides each unique to female (COL1A1 and COL5A2) or male (SAA1 and KRT 9) patients were selected and mouse homologs of the peptides were screened for self-reactive immune responses in apoE–/– mice. The screening step revealed potential sex-influenced immune responses which was associated with differential immune profiles. Based on the frequency in patient plasma, COL6A1, COL5A2, and KRT 9 peptides were then tested in immunization studies. Neither COL5A2 nor KRT 9 peptide immunization resulted in significant effects on atherosclerosis compared to controls. On the other hand, female mice immunized with COL6A1 peptide had significantly reduced atherosclerosis whereas male mice had significantly increased atherosclerosis, associated with differential immune profiles. Our study identified potential self-antigens involved in atherosclerosis using the immune peptidome of CAD patients. Altering self-reactive immune responses to COL6A1 in apoE–/– mice resulted in differential effects on atherosclerosis burden with sex as a determinant of outcome.

## Introduction

Inflammation (1) and adaptive immune responses (2) are implicated in coronary artery disease (CAD). Sex-influenced differential pathobiology in CAD (3) may be associated with sexual dimorphism of immune responses (4–6). The hallmark of adaptive immune responses is antigen-specificity and key to understanding the immune response to atherosclerosis is the identification of relevant antigens. One potential approach to identify antigens is to investigate specific HLA types relevant in CAD and then characterize the peptides bound to the specific HLA. However, there is at best weak associations between specific HLA types and CAD (7). A CAD risk allele was identified within the MHC locus in close proximity to class-I HLA genes yet no common HLA types accounted for the association (8). An alternative strategy would be to identify potentially common disease-relevant antigens independent of HLA type. This would be inclusive of various HLA types since an antigen is not necessarily limited to a single HLA (9). Assuming that there are antigens common among CAD patients, some of these are potentially self-peptides given the chronic nature of CAD (10–12). Self-peptides are presented by class-I HLA as part of normal cellular homeostasis and is involved in immune surveillance (13). Because normal immune surveillance function is dependent on self-antigen recognition by the immune system, alterations in class-I HLA peptide profiles have been used to identify potential antigens involved in diseases (9). This is may be of significance given the CAD risk allele in close proximity to class-I HLA genes (8). It is notable that sex bias occurs in class-I HLA associated immune responses (14).

Atherosclerosis is the major underlying cause of CAD. We have reported that immune precipitation of soluble class-I HLA may be used to identify potential atherosclerosis self-antigens (15). However, the report was limited to a small number of male patients. To increase the scope and robustness of the investigation, the study was expanded to include female patients and used two different MS/MS methods to increase the rigor of the approach. The objective of the study was to investigate commonality and differences between female and male sex in self-antigens potentially involved in the self-reactive immune responses in CAD. The role of the immune responses to the self-peptides were assessed in immunization studies of apoE–/– mice.

## Materials and Methods

### Plasma Samples

Patient plasma samples were obtained from frozen aliquots of a previously completed IRB-approved study called Azithromycin in Acute Coronary Syndrome (AZACS) (16). Samples from 9 female and 11 male patients aged 50 years and over were used under an IRB protocol (Pro00034283) which limited the approved use of patient data for this study to age and sex only. Patients were classified as unstable angina (chest pain/discomfort consistent with myocardial ischemia at rest lasting for at least 5 min and electrocardiographic evidence of ischemia, new wall motion abnormalities, or evidence of coronary artery disease) or acute myocardial infarction (chest pain/discomfort lasting at least 5 min, with one episode within 24 h of admission, raised CKMB or troponin I, or new or presumed new Q-waves in at least two contiguous leads) (16). Plasma samples from self-reported controls aged 50 years and over, 10 of each sex, were purchased (Innovative Research). Mean ages were Patient: 63 ± 8.8; Control: 56.5 ± 4.6; *P* < 0.05.

### Immuno-Precipitation and UPLC-MS/MS

Immuno-precipitation of soluble HLA/peptide complexes were performed as described (15). Capture antibody to HLA–A, –B, and –C (clone W6/32) was conjugated to agarose beads using a commercially available kit (AminoLink Plus Coupling, Thermo Fisher) and added to plasma diluted 10x in TBS buffer with 0.01% Silent Surfactant (Expedion), rotated for 18 h in 4°C. The samples were then heat denatured at 95°C for 10 min, cooled, and loaded in size-exclusion centrifugation columns cut-off at 3kD (Amicon). The filtrate containing the peptides were then used for ultraperformance liquid chromatography-tandem mass spectrometry (UPLC-MS/MS).

The peptides were analyzed by the Cedars-Sinai Mass Spectrometry and Biomarker Discovery Core. Peptides were desalted by C_18_-Stage Tips concentrated in a Speed Vac concentrator, and reconstituted in 25 μL 0.2% formic acid. Ten μL peptide solution was injected and loaded on a trap column (75 μm × 2 cm, C_18_), separated on an EASY-Spray analytical column (PepMap^TM^ RSLC C18, 2 μm, 100Å, 50 μm × 15 cm), and analyzed by an LTQ Orbitrap Elite hybrid mass spectrometer (Thermo Fisher) operated in the positive ion mode essentially as described (17). Mass spectra were acquired in a data-dependent manner, with automatic switching between MS and MS/MS scans. In MS scans, the lock mass at *m/z* 445.120025 was applied to provide internal mass calibration (18). For MS/MS scans with higher sensitivity, up to 20 most intense peaks with charge state ≥2 were automatically selected for fragmentation by rapid collisional-induced dissociation (rCID). For MS/MS scans with higher accuracy, up to 15 most intense peaks with charge state ≥2 were automatically selected for MS/MS fragmentation by higher-energy collisional dissociation (HCD). The acquired MS data was searched against the Uniprot_Human database (released on 02/20/2014, containing 88,647 sequences) using the Andromeda algorithm (19) in the MaxQuant (v1.3.0.5) environment (20). The MS/MS peaks were deisotoped and searched using a 0.5 Da mass tolerance for the rCID dataset or a 20 parts-per-million (ppm) mass tolerance for the HCD dataset.

### Self-Peptide Selection

Peptides found only in patient samples and identified by both the rCID and HCD methods in at least one patient were considered patient-unique peptides and ranked according to frequency then sub-grouped further as common to both sexes or unique to either sex. Mouse homologs of the peptides were searched using BLAST (PubMed). The mouse peptide sequences were flanked on each side with the corresponding peptides to increase potential binding to mouse MHC-I since immunologically reactive peptides from homologous proteins may differ between humans and mice (15). The flanked peptide sequences were then assessed for potential binding to mouse MHC-I using the epitope binding score prediction tool on the Immune Epitope Database (IEDB) website (15, 21). Epitope length for analysis was set at 8-mer for H2K^b^ and 9-mer for H2D^b^. For low-scoring peptides, additional amino-acids were added corresponding to the published sequence until epitope prediction scores improved (15). Peptides were then synthesized at >95% purity (LifeTein).

### Animals

The studies were approved by the Cedars-Sinai Institutional Animal Care and Use Committee. Female and male apoE–/– mice were purchased from Jackson Lab at 6 weeks of age. For the peptide screening study, mice of both sexes were fed normal chow or high fat diet consisting of 21% fat, 0.15% cholesterol (TD.88137, Envigo) for 6 weeks starting at 7 weeks of age and euthanized at 13 weeks of age. Spleens were collected for *in vitro* peptide stimulation. Potential sex-influenced differences in atherosclerosis and immune responses were characterized in mice of both sexes fed high fat diet starting at 13 weeks of age until euthanasia at 25 weeks of age.

### Peptide Screening

Peptides selected for screening as potential self-antigens were resuspended at a concentration of 20 μg/ml in RPMI 1,640 medium supplemented with 10% FBS and antibiotic/antimycotic (11, 15). Splenocytes from 13 week-old mice of both sexes fed either normal chow or high fat diet were collected and subjected to RBC lysis. Cells were then pooled according to sex and diet, counted and aliquoted for peptide stimulation in triplicates. One aliquot (1 × 10^6^/0.15 ml) was incubated with a specific peptide for 1 h and then transport inhibitor Monensin was added for an additional 5-h incubation in 37°C/5% CO_2_ for intracellular cytokine staining. Intracellular staining was performed using fixation and permeabilization steps. Another aliquot was incubated with a specific peptide for 24 h to profile Central Memory and Effector Memory T cells (11, 15). See Table S1 for specific antibodies.

### Immunization

Mice fed normal chow were grouped into 1 of 3 immunization studies according to the appropriate sex: immunization of both sexes with a peptide common to both sexes, immunization of females with a female-unique peptide, or immunization of males with a male-unique peptide. Immunization was performed using subcutaneous injection of 20 μg of peptide formulated in Adjuvant consisting of Adju-Phos (Brenntag; 12.5 μl of a 2% solution) and 10 μg MPLA (MPLA-SM VacciGrade, InvivoGen) in a volume of 200 μl (11). Control was injection with Adjuvant alone. Immunizations were performed at 7, 10, and 12 weeks of age (11, 22). Mice were fed high fat diet at 13 weeks of age until euthanasia at 25 weeks of age. Serum was collected for antibody and cholesterol levels. Aortas were processed in Histochoice (Amresco) and stained with Oil Red-O for en face analysis. Hearts were harvested and embedded in OCT compound (Tissue-Tek). Ten-micron cryo-sections of the aortic sinus were collected. Lipid was stained using Oil-Red-O. Staining for macrophage was performed using MOMA-2 antibody. Three slides of approximately 0.1 millimeter intervals for each animal were used for each stain and averaged. Image analysis was performed using ImagePro (Media Cybernetics). Spleens were collected for T cell profiling. Briefly, spleens were manually disrupted to release splenocytes and subjected to RBC lysis. An aliquot was then incubated with 1x Monensin in RPMI 1,640 medium with antibiotic/antimycotic for 4 h in 37°C/5% CO_2_ for intracellular cytokine staining. For degranulation assay to measure CD8+ T cell cytolytic activity, splenocytes were incubated with peptide (20 μg/ml) and 2.5 μg/ml fluorescent conjugated CD107a for 1 h followed by the addition of Monensin and incubation for another 4 h (11, 22). Cells were collected and stained for flow cytometry. Another aliquot of splenocytes was incubated for 48 h with the specific peptide (20 μg/ml) used for immunization and then subjected to surface staining for Central Memory and Effector Memory T cells. A small portion of the spleen was flash-frozen for mRNA analysis.

### Serum IgG and IgM

Mouse serum was diluted 1:50 and 1:100 with 1X PBS for the detection of IgG and IgM against peptide, respectively, by ELISA using standard protocol on Maxisorp 96 well-plates coated with peptide (LifeTein) at 20 μg/ml in Na_2_CO_3_-NaHCO_3_ buffer, pH 9.6. Two percent goat serum in 1X PBS was used for blocking and dilution of biotin-conjugated anti-mouse IgG (Thermo Scientific) or IgM (SouthernBiotech) antibodies in 1:5000 and 1:10000 weight to volume dilutions, respectively. Detection was with avidin-HRP (eBiosciences) and ABTS substrate (SouthernBiotech) analyzed at 405 nm.

### Ctla4, Pdcd1, and IL-1β mRNA

Total RNA was extracted from mouse spleens with TRIzol reagent (Life Technologies) and reverse transcribed using SuperScript VILO cDNA Synthesis Kit (Invitrogen). Quantitative real-time PCR was performed using iTaq Universal SYBR Green Supermix and iQ5 Real-Time PCR Detection System (Bio-Rad). GAPDH was used as reference gene. The data was analyzed by the Ct_ΔΔ_ method using one Adjuvant control sample as calibrator and presented as fold change relative to the calibrator.

### IL-1β ELISA

Mouse serum IL-1β levels were measured using an ELISA kit (eBioscience) according to manufacturer's instructions. Samples out of detection limit of the standard curve were excluded from analysis.

### Statistics

UPLC-MS/MS peptides with a false discovery rate (FDR) of ≤ 1% were accepted. Posterior Error of Probability (PEP) was used to assess statistical significance of individual peptides. Pearson correlation coefficient analyzed rCID and HCD correlations. Other data are presented as mean ± standard deviation. Normally distributed data of multiple group experiments were analyzed by ANOVA. For analysis of peptide screening data, Dunnett's test was used with no peptide stimulation as control. For other experiments with multiple group comparisons, ANOVA was followed by Holm-Sidak test. Non-normally distributed data of multiple group studies were analyzed by Kruskal–Wallis test followed by Dunn's multiple comparisons test. Unpaired *t*-test was used for analysis of two groups with normally distributed data; non-normally distributed data of two groups were analyzed by Mann–Whitney test. Statistical significance was considered at *P* < 0.05 but data trends were also noted.

## Results

### Validation of IP and MS/MS Process

**Figure 1:**
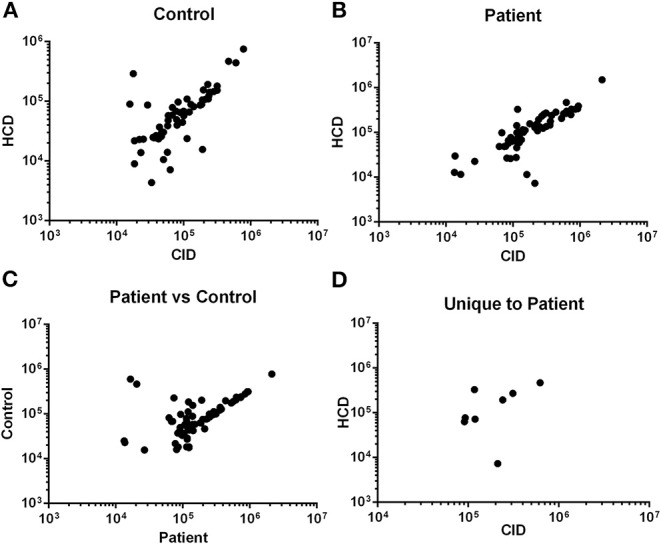
Validation of MS/MS approach. Signal intensity of peptides from rapid collisional-induced dissociation (rCID) method was plotted against signal intensity of identical peptides from high energy collisional dissociation (HCD) method in Control [**(A)**; Pearson *R*^2^ = 0.790; *P* < 0.0001] or Patient [**(B)**; Pearson *R*^2^ = 0.849; *P* < 0.0001] plasma sample. Correlation between signal intensity of peptides common to Control and Patient samples from rCID method [**(C)**; Pearson *R*^2^ = 0.706; *P* < 0.0001]. Signal intensity of peptides unique to patient detected by both rCID and HCD methods [**(D)**; Pearson *R*^2^ = 0.745; *P* = 0.034].

To increase the robustness and stringency of the approach compared to our previous report (15), two different MS/MS fragmentation methods were used. Peptides identified from rCID fragmentation were compared to HCD fragmentation and the signal intensity of peptides common to both methods was used for validation. There was significant correlation in the signal intensity of peptides detected by both methods in a control sample (Figure 1A) and in an ACS sample (Figure 1B). Peptides common to both the control and the ACS sample detected by rCID were also significantly correlated (Figure 1C), as were the peptides detected by both rCID and HCD methods that were unique to the ACS sample (Figure 1D), validating the use of both MS/MS methods to increase the stringency and robustness of the process.

### Patient-Unique Self-Peptides

Selection of patient-unique peptides identified by MS/MS is described in the Methods section. Peptide length was not considered in the selection process given the existence of antigens with non-canonical peptide lengths (23, 24), and protein interaction with class-I HLA (24, 25) of which the immunologic functions remain to be clarified. Selected patient-unique peptides were then ranked according to frequency as determined by the MS/MS methods (Tables S2–S4), and further classified as common to both males and females, or unique to one sex (Table 1). The top 3 patient peptides common to males and females (COL6A1, CDSN, SAA2), as well as the 2 female patient-unique peptides (COL1A1, COL5A2), and the top 3 male patient-unique peptides (SAA1, SAA2, KRT9) with a PEP <0.05 were selected for further study.

**Table 1:** Patient-unique peptides grouped as common to both sexes, or specific to sex, and ranked by frequency (peptide sequences are found in Supplemental Material).

**Protein name**	**# Male**	**# Female**	**Combined**	**Frequency (% of patients)**	**Posterior error of probability**
**Common to both sexes of patients**					
COL6A1	8	5	13	65	1.3 × 10^−69^
CDSN	4	1	5	25	5.13 × 10^−20^
SAA2	4	1	5	25	1.03 × 10^−11^
SAA1	4	1	5	25	0.0004
SAA1	2	1	3	15	0.0639
**Unique to female patients**					
COL1A1	0	2	2	22	2.2 × 10^−8^
COL5A2	0	2	2	22	0.0083
**Unique to male patients**					
SAA1-4	3	0	3	27	1.87 × 10^−5^
SAA1;SAA2	3	0	3	27	0.04133
KRT9	2	0	2	18	2.51 × 10^−38^
DMKN	2	0	2	18	0.00477
ANK1	1	0	1	9	0.00211
KLC3	1	0	1	9	0.02990
MRVI1	1	0	1	9	0.03181

### Mouse Homologs and MHC-I Binding Prediction

Mouse homologs of the selected peptides were searched using BLAST to test the potential role in the immune response during atherosclerosis development in apoE–/– mice. The peptide for SAA2 common to both sexes flanked the SAA2 peptide unique to males in the homologous mouse sequence. Thus, the mouse SAA2 peptide was extended to capture both peptides for the rest of the studies. Furthermore, to account for potential differences in the MHC-I binding properties between human and mouse, the homologous mouse peptides were assessed for H2K^b^ and H2D^b^ binding as predicted by the online tool IEDB (21). Peptides with low prediction scores were flanked on each amino and carboxy terminals with the respective mouse sequence until predicted binding scores improved to at least 20th percentile. The final peptide sequences used are shown in Table 2. Note that the CDSN peptide fragment with good MHC-I binding prediction score was in close proximity to but did not include the homologous peptide. The peptides were then synthesized for use in the mouse experiments.

**Table 2:** Homologous mouse peptide sequence used for screening self-reactive responses.

**Name**	**Sequence**
COL6A1*	GLNGTKGYPGLKGDEGEVGDPGEDNNDISPRGVKGAKGYRGPEGP
	QGPPG
CDSN	SSSGSLIYKPGTGYSQSSYSYGSGGSRPGG
SAA2	QEFFGRGHEDTMADQEANRHGRSGKDPNYYRPPGLPDKY
COL1A1	DGEAGAQGAPGPAGPAGERGEQGPAGSPGFQGLPGP
COL5A2	RGAPGKDGEVGPSGPVGPPGLAGERGEAGP
SAA1	AGDMWRAYTDMKEANWKNSDKYFHARGNYD
KRT9	EKNALTKNHKEEMSQLTGQNDGDVNVEINVAPST

Text underlined are corresponding regions of mouse sequence homologous to the human peptides, text without underline are flanking peptides according to the reported mouse sequence. *Region of homology did not show high binding prediction score for mouse MHC-I binding, so peptide was flanked until binding prediction score was increased.

### Differential Self-Reactive Immune Response in apoE–/– Mice

**Figure 2:**
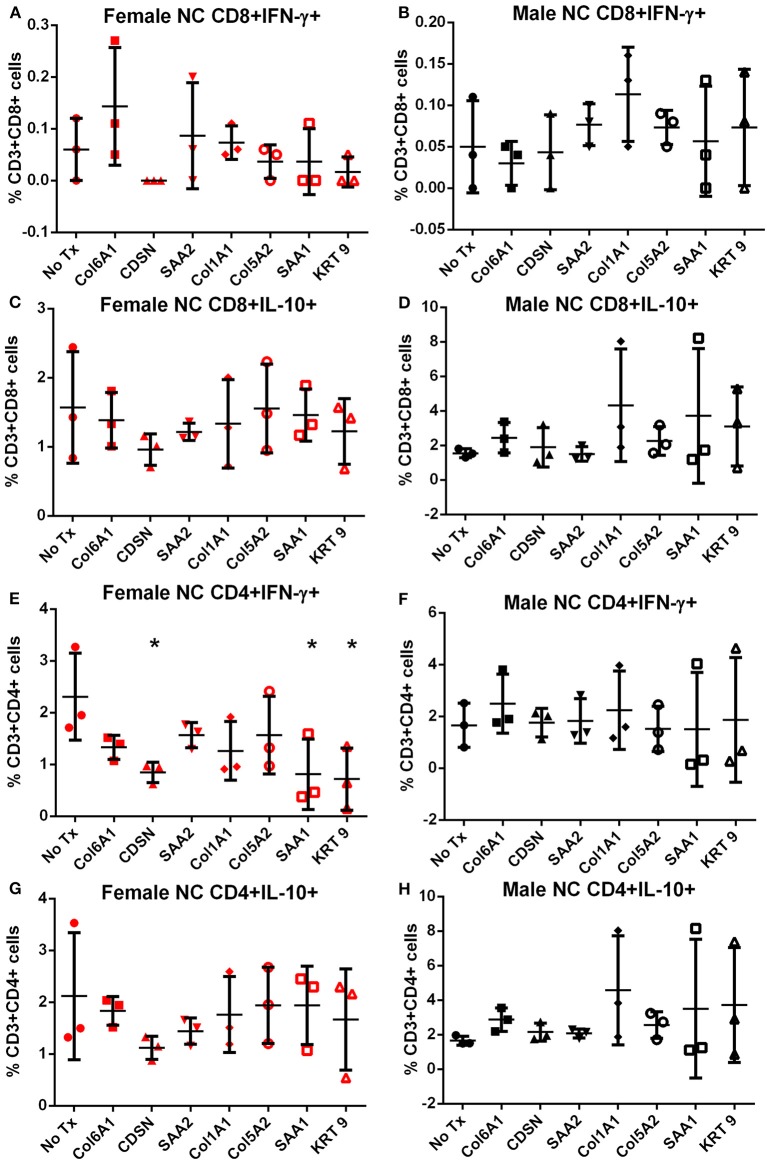
Intracellular cytokine staining of splenocytes from mice fed with normal chow. Splenocytes from female **(A,C,E,G)** or male **(B,D,F,H)** apoE–/– mice fed normal chow (NC) stimulated for 6 h with mouse homologs of self-peptides selected from patient immune peptidome and stained for intracellular IFN-γ and IL-10. *N* = 3 each; *P < 0.05 compared to No Tx. Gating scheme in Figure S1.

**Figure 3:**
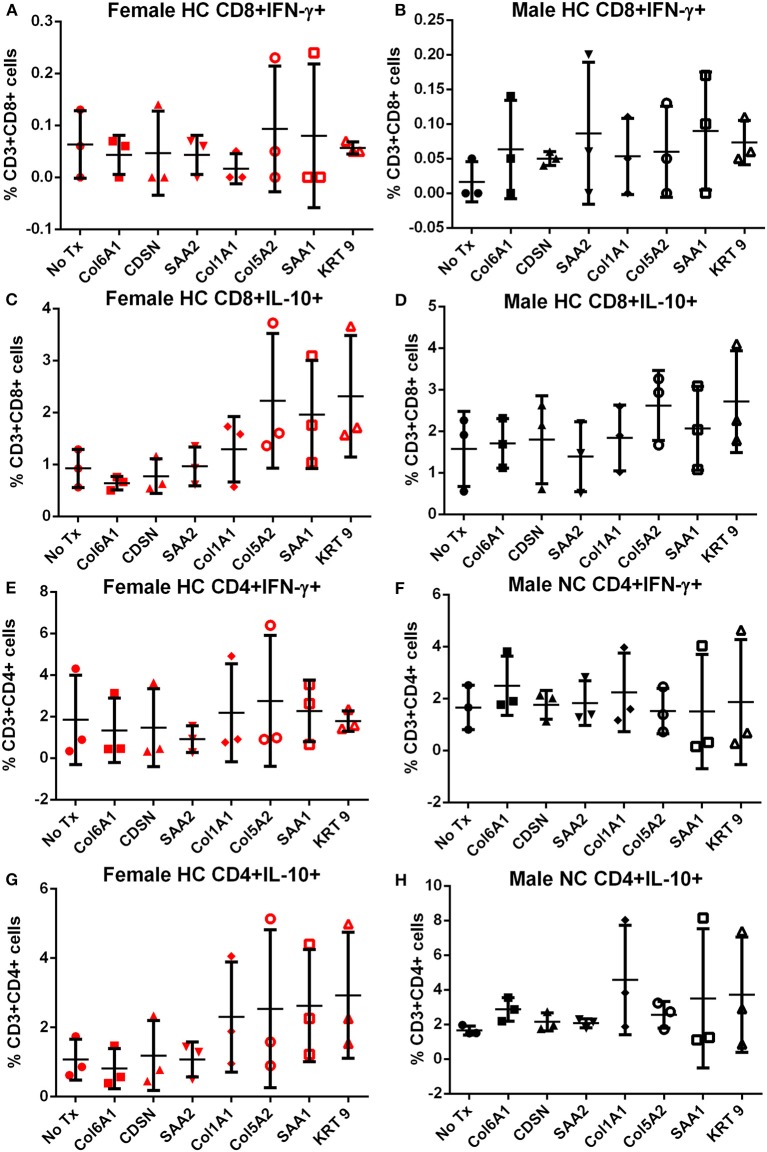
Intracellular cytokine staining of splenocytes from mice fed with high fat diet. Splenocytes from female **(A,C,E,G)** or male **(B,D,F,H)** apoE–/– mice fed high fat diet (HC) stimulated for 6 h with mouse homologs of self-peptides selected from patient immune peptidome and stained for intracellular IFN-γ and IL-10. *N* = 3 each. Gating scheme in Figure S1.

Splenocytes from female and male apoE–/– mice fed normal chow or high fat diet for 6 weeks were used to screen the 7 peptides selected to test for self-reactive immune responses (15). There was no significant difference in intracellular cytokine stain of T cells among the groups tested after 6 h of peptide stimulation (Figure S1 and Figures 2, 3) with the exception of female mice fed normal chow, which had decreased CD4+IFN-γ+ T cells after stimulation with CDSN, SAA1, or KRT 9 (Figure 2E).

**Figure 4:**
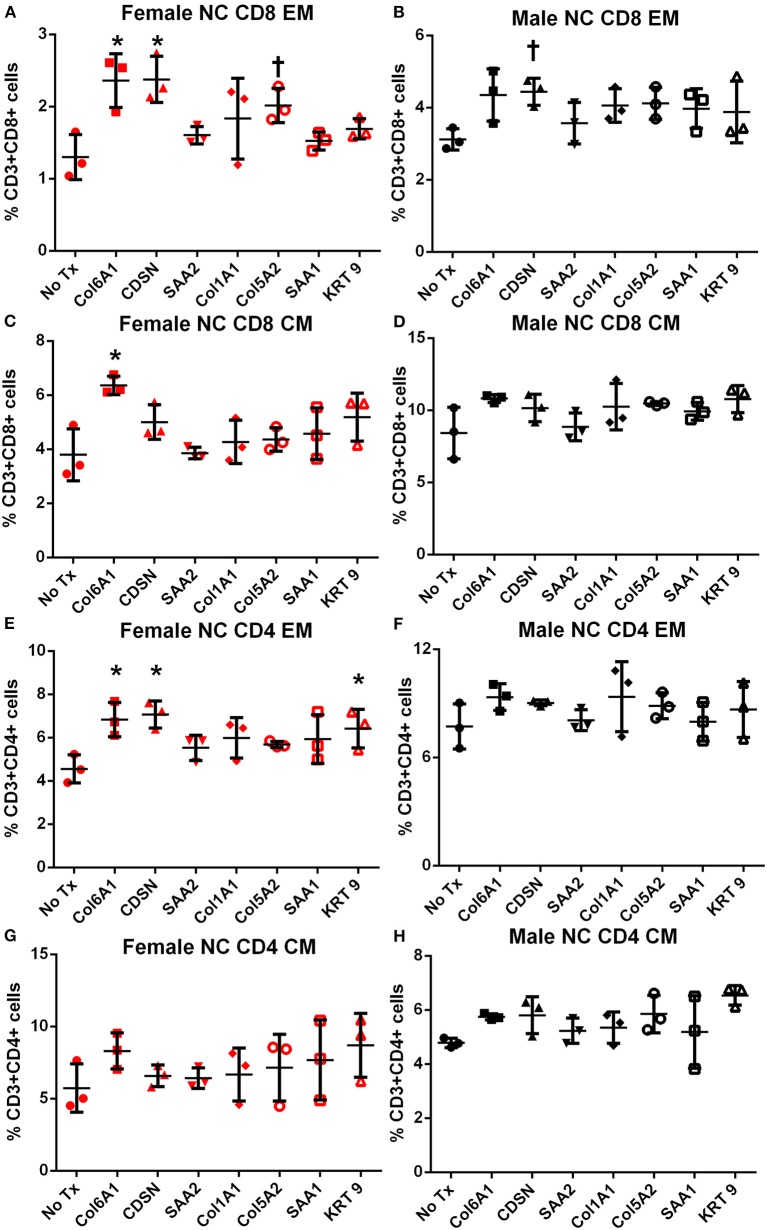
Memory T cell response to peptide stimulation of splenocytes from normal chow-fed mice. Splenocytes from female **(A,C,E,G)** or male **(B,D,F,H)** apoE–/– mice fed normal chow (NC) for 6 weeks stimulated with mouse homologs of self-peptides or no stimulation (No Tx) for 24 h and then stained for Effector Memory (EM) or Central Memory (CM) T cells. *N* = 3 each; *P < 0.05, *P* = 0.06 compared to No Tx. Gating scheme depicted in Figure S2.

Memory T cell response was assessed in splenocytes stimulated with the individual peptides for 24 h. In female mice fed normal chow, there was significantly increased CD8+ and CD4+ Effector Memory (EM) T cell response to COL6A1 and CDSN (Figure S2 for gating scheme; Figures 4A,E, respectively), a trend for increased CD8+ EM response for COL5A2 (Figure 4A), and increased CD4+ EM response to KRT 9 (Figure 4E). Central Memory (CM) response remained unchanged after peptide stimulation except for COL6A1 which increased CD8+ CM response (Figure 4C). In male mice fed normal chow, CD8+EM T cells were trending higher in response to CDSN (Figure 4B)

**Figure 5:**
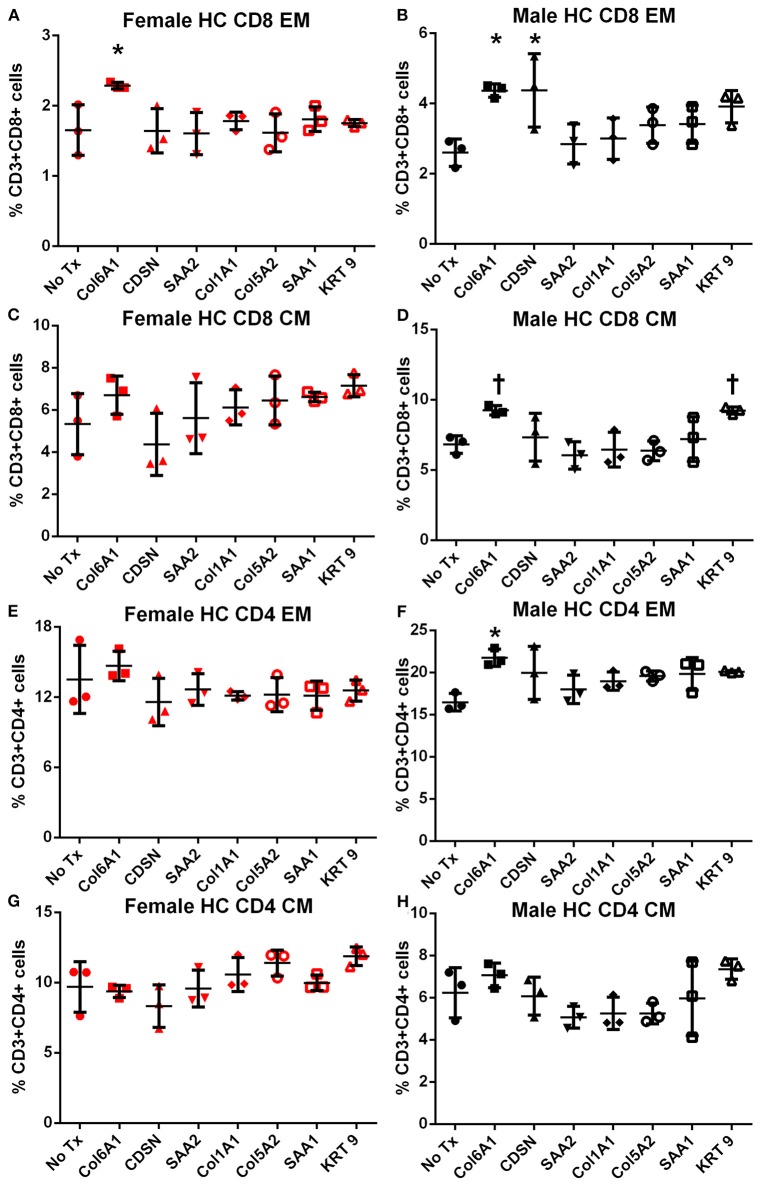
Memory T cell response to peptide stimulation of splenocytes from high fat diet-fed mice. Splenocytes from female **(A,C,E,G)** or male **(B,D,F,H)** apoE–/– mice fed high fat diet (HC) for 6 weeks stimulated with mouse homologs of self-peptides or no stimulation (No Tx) for 24 h and then stained for Effector Memory (EM) or Central Memory (CM) T cells. *N* = 3 each; *P < 0.05, *P* = 0.06 compared to No Tx. Gating scheme depicted in Figure S2.

In female mice fed high fat diet, CD8+ EM T cells were increased in response to COL6A1 (Figure 5A). Male mice fed high fat diet had significantly increased CD8+ EM response to COL6A1 and CDSN (Figure 5B), and CD4+ EM response to COL6A1 (Figure 5F). There was also a trend for increased CD8+ CM response to COL6A1 and KRT 9 (Figure 5D) in male mice fed high fat diet. Based on the collective results of the screening test and with the aim to test one peptide common to patients of both sexes and one peptide each unique to male or female patients, COL6A1 (common), COL5A2 (female), and KRT 9 (male) were selected for immunization studies. The differential response to individual peptide stimulation between female and male mice also suggested that there were inherent differences in the immune profile between female and male apoE–/– mice that may influence atherosclerosis. To assess if there was an association between the differential immune response to the self-peptides observed and atherosclerosis, we examined atherosclerosis in female compared to male apoE–/– mice fed high fat diet for 12 weeks.

### Atherosclerosis and Sex-Influenced Immune Regulation

**Figure 6:**
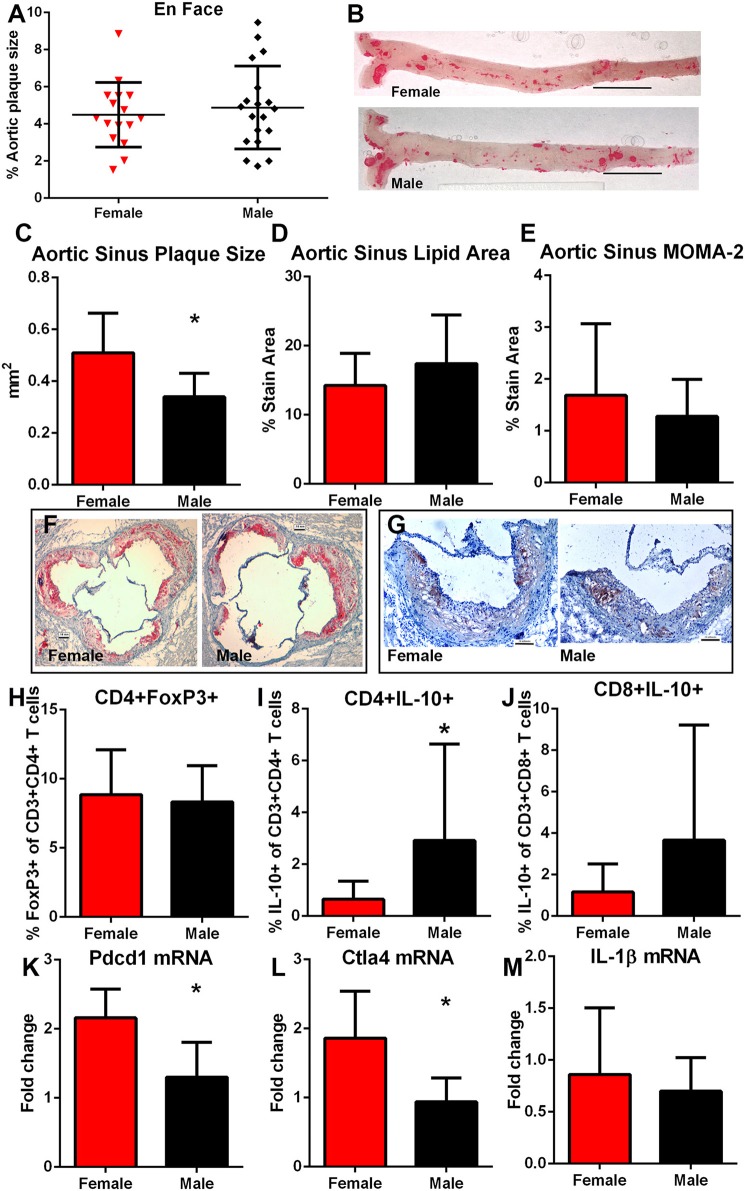
Influence of sex on atherosclerosis and immune regulation. Atherosclerotic burden assessed by en face Oil Red-O staining in aortas of female (*N* = 20) and male (*N* = 17) apoE–/– mice **(A)** fed high fat diet for 12 weeks starting at 13 weeks of age [representative photographs in **(B)**; bar=0.5 cm]. Aortic sinus plaque size **(C)**, lipid area **(D)**, and macrophage stain area **(E)** measurements with representative photographs of Oil red-O stain **(F)** and MOMA-2 stain **(G)**. Bar = 0.1 mm. Immune response regulators CD4+FoxP3+ regulatory T cells [**(H)**, female *N* = 19, male *N* = 17], Type-2 CD8+ **(I)** and CD4+ **(J)** T cells expressing IL-10 (female *N* = 19, male *N* = 17) in splenocytes, and splenic mRNA expression of Pdcd1 [**(K)**, *N* = 5 each], Ctla4 [**(L)**, *N* = 5 each], and IL-1β [**(M)**, *N* = 5 each). *P < 0.05, *T*-test for **(C,K,L)**; Mann–Whitney test for **(I)**. Gating for flow cytometric analysis of intra-cellular stain as depicted in Figure S1.

After 12 weeks of high fat diet, there was no significant difference in percent aortic plaque area in female compared to male mice (Figures 6A,B) even as there was significantly lower serum cholesterol in female compared to male mice (1397 ± 237.6 mg/dL *N* = 20 vs. 1939±281.8 mg/dL *N* = 17, respectively; *P* < 0.01). Body weight was also significantly lower in female compared to male mice (30.3 ± 3.9 vs. 43.5 ± 5.1 gm, respectively; *P* < 0.01). Aortic sinus plaque size was bigger in female mice compared to male mice (Figures 6C,F), but no differences in lipid area or macrophage presence were noted (Figures 6D–G). Given the observed differential immune response to the self-peptides between female and male mice observed (Figures 4, 5), we evaluated potential pathways involved in regulating immune responses to self-antigens which include CD4+FoxP3+ T_reg_ cells, Type-2 cytokine T cells, and immune checkpoints. There was no significant difference in CD4+FoxP3+ T_reg_ cells between female and male mice (Figure 6H). CD4+IL-10+ T cells (Figure 6I) were significantly increased in male mice compared to female mice and CD8+IL-10+ T cells were trending higher (Figure 6J; *P* = 0.09). On the other hand, there was significantly higher Pdcd1 (Figure 6K) and Ctla4 (Figure 6L) mRNA expression in female compared to male mice. Despite potential differences in immune regulatory pathways, no differences were detected in IL-1β mRNA expression (Figure 6M). Thus, the results suggested inherently differential profiles of immune regulation between female and male apoE–/– mice even as aortic atherosclerosis was comparable. The self-antigens were then used in immunization studies as immune challenge to assess effects on atherosclerosis.

### Sex as Determinant of Outcome After Immunization

**Figure 7:**
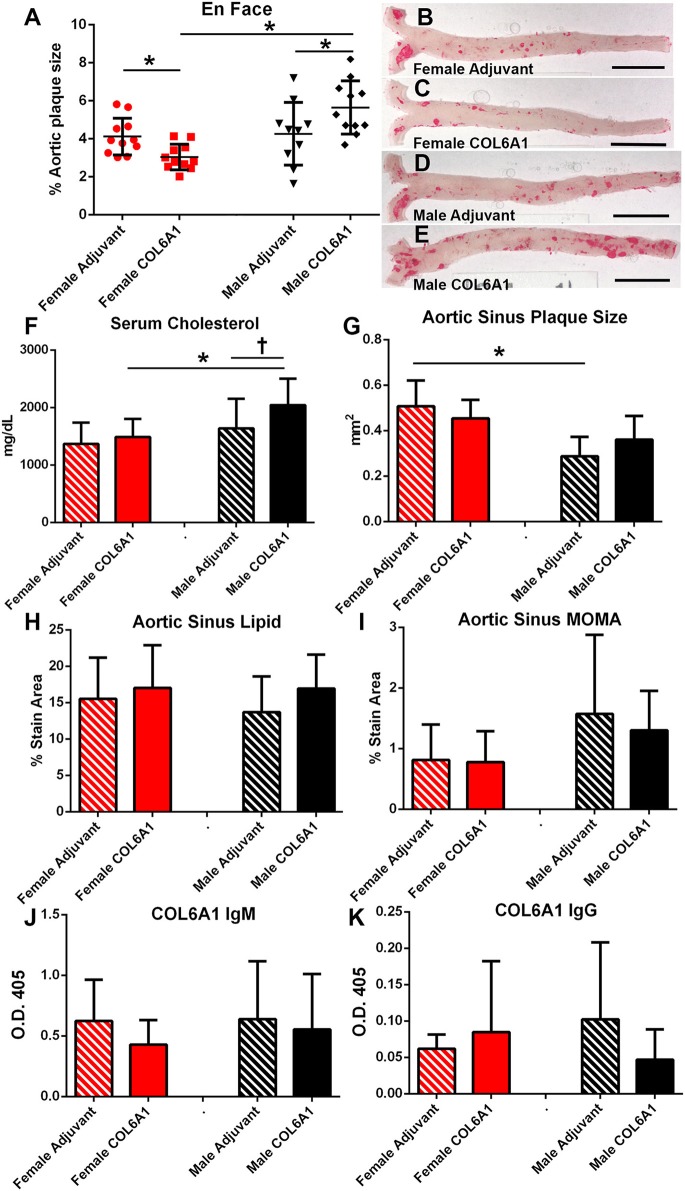
Sex as determinant of outcome in COL6A1 immunized apoE–/– mice. Female and male apoE–/– mice were immunized with the mouse homolog of collagen 6A1 (COL6A1) and effect on atherosclerosis was measured on en face Oil Red-O staining of the aorta **(A)**. *P < 0.05 by ANOVA and Holm–Sidak's post–test. Representative photographs of en face Oil Red-O preparation of aortas from female mice injected with Adjuvant alone **(B)** or COL6A1 peptide **(C)** and male mice injected with Adjuvant alone **(D)** or COL6A1 **(E)**. Bar = 0.5 cm. Female mice *N* = 11 each; Male Adjuvant *N* = 10, Male COL6A1 *N* = 11. Serum cholesterol **(F)**, aortic sinus plaque size **(G)**, aortic sinus lipid as stained with Oil Red-O **(H)**, and sinus plaque macrophage presence as stained with MOMA-2 **(I)** in mice immunized with COL6A1. Anti-COL6A1 IgM levels **(J)** and anti-COL6A1 IgG levels **(K)**. *P < 0.05; *P* = 0.065; *N* = 7–11 per group.

COL6A1, COL5A2, and KRT 9 peptides were selected for immunization studies in apoE–/– mice to test if self-reactive immune responses and atherosclerotic burden may be altered when challenged with self-antigens. Based on the result of the IP-MS/MS assay, COL5A2 which was unique to female patients was used to immunize female mice; KRT 9 which was unique to male patients was used to immunize male mice; and COL6A1 which was common to majority of both male and female patients was used to immunize both male and female mice. Adjuvant injection itself (Figures 7A–E) had no effect on aortic atherosclerosis when compared to age- and sex-matched naïve mice shown in Figure 6A.

There was differential effect on atherosclerosis in mice immunized with COL6A1, wherein female mice had significantly reduced while male mice had significantly increased aortic atherosclerosis (Figures 7A–E). Serum cholesterol was higher in COL6A1 immunized male mice (Figure 7F). Aortic sinus plaques were not significantly changed by COL6A1 immunization but the differential aortic sinus plaque size between females and males was again observed in the Adjuvant groups (Figure 7G). No differences were observed in aortic sinus plaque lipid and macrophage stain among the groups (Figures 7H,I, respectively). Serum anti-COL6A1 IgM and IgG were not different among the groups (Figures 7J,K, respectively).

**Figure 8:**
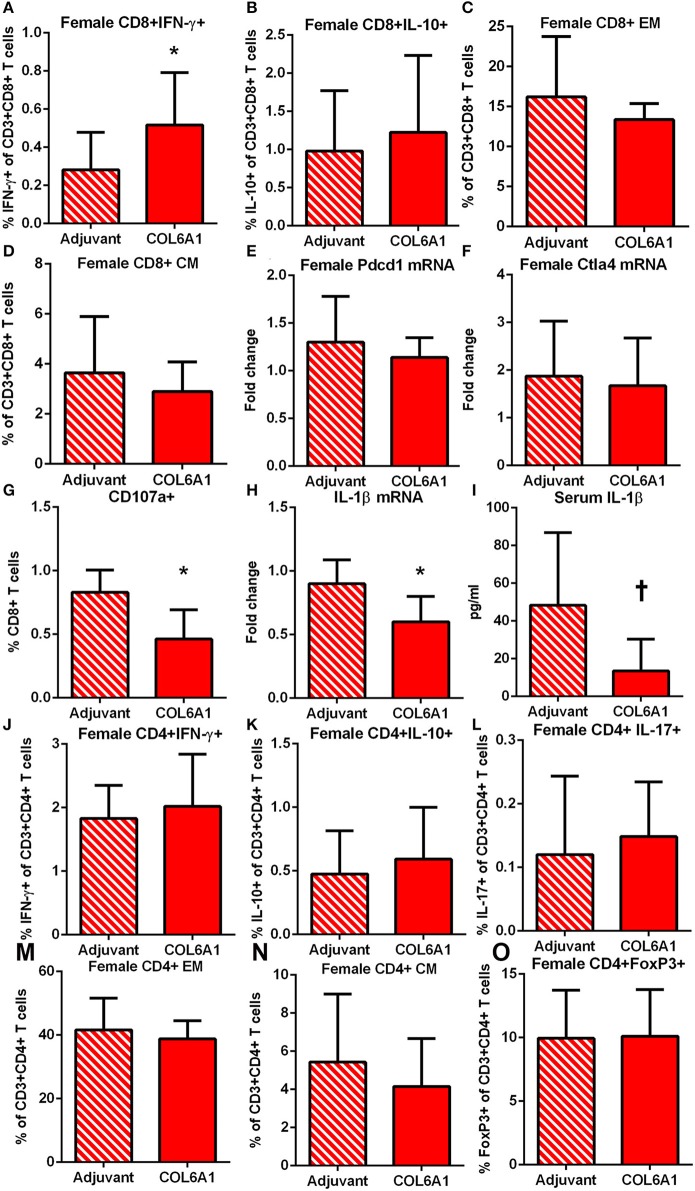
Immune profile of female apoE–/– mice immunized with COL6A1. Intracellular staining of splenocytes from female mice immunized with COL6A1 [**(A,B)**; *N* = 11 each]. Splenocytes were also stimulated with COL6A1 peptide for 48 h and stained for Effector Memory (EM) and Central Memory (CM) CD8+ T cells [**(C,D)**, respectively; *N* = 6–7 each]. Pdcd1 and Ctla4 mRNA expression in spleens [**(E,F)**; *N* = 5 each]. Cytolytic activity of CD8+ T cells assessed by CD107a stain in splenocytes of female mice after 5 h stimulation with COL6A1 peptide [**(G)**; *N* = 4–5 each]. IL-1β mRNA expression in spleens [**(H)**; *N* = 5 each] and serum IL-1β [**(I)**; *N* = 5 each]. *P < 0.05, *P* =0.055. Intracellular staining of CD4+T cell **(J–L)**, EM and CM CD4+ T cells [**(M,N)**, respectively], and CD4+FoxP3+ T cells **(O)**. Gating schemes for flow cytometric analysis as depicted in Figure S1 for cytokine stain, Figure S2 for Memory cells, Figure S3 for CD107a, and Figure S4 for CD4+IL-17+ T cells.

The immune response to COL6A1 immunization in female mice was characterized by increased CD8+IFN-γ+ T cells (Figure 8A) but no changes in CD8+IL-10+ T cells (Figure 8B), nor were there any changes in EM or CM T cell response (Figures 8C,D). Pdcd1 and Ctla4 mRNA expression were unchanged (Figures 8E,F). Cytolytic activity of CD8+ T cells measured by CD107a degranulation assay (Figure 8G and Figure S3) was decreased in COL6A1 immunized mice. Splenic IL-1β mRNA expression (Figure 8H) and serum IL-1β (Figure 8I) of immunized female mice were also reduced. CD4+ T cell responses (Figures 8J–N) and CD4+FoxP3+ T cells (Figure 8O) were not different.

**Figure 9:**
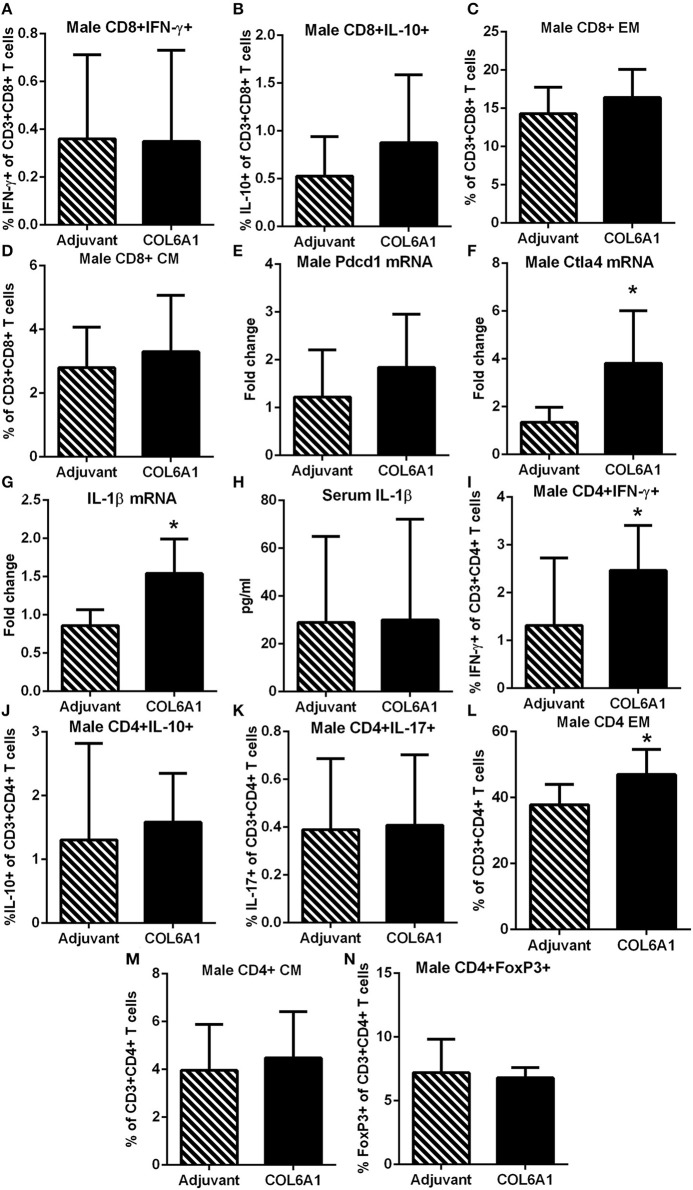
Immune profile of male apoE–/– mice immunized with COL6A1. Intracellular staining of CD8+ T cells in splenocytes from male mice immunized with COL6A1 [**(A,B)**; *N* = 7–11 each]. Splenocytes were also stimulated with COL6A1 peptide for 48 h and stained for Effector Memory (EM) and Central Memory (CM) CD8+ T cells [**(C,D)**, respectively; *N* = 7–8 each]. Pdcd1 and Ctla4 mRNA expression in spleens of male mice [**(E,F)**; *N* = 5 each]. IL-1β mRNA expression in spleens [**(G)**; *N* = 5 each] and IL-1β in serum [**(H)**; Adjuvant *N* = 4, COL6A1 *N* = 7]. Intracellular staining of CD4+T cells **(I–K)**, EM and CM CD4+ T cells [**(L,M)**, respectively], and CD4+FoxP3+ T cells **(N)**. *P < 0.05; *T-*test. Gating scheme for flow cytometric analysis as depicted in Figure S1 for cytokine stain, Figure S2 for Memory T cells and Figure S4 for CD4+IL-17+ T cells.

Male mice on the other hand had no differences observed in CD8+ T cell response (Figures 9A–D). Pdcd1 mRNA was unchanged, but Ctla4 mRNA was significantly increased (Figures 9E,F, respectively) in COL6A1 immunized male mice. Splenic IL-1β mRNA expression (Figure 9G) in immunized male mice was significantly increased but serum IL-1β was not different (Figure 9H). There was increased CD4+IFN-γ+ T cells (Figure 9I) with no differences in CD4+IL-10+ or CD4+IL-17+ T cells (Figures 9J,K, respectively; Figure S4). CD4+ EM T cells (Figure 9L) increased, but no change in CD4+ CM T cells (Figure 9M) and CD4+FoxP3+ T cells (Figure 9N) were noted. There were no significant effects on atherosclerosis or immune profile when mice were immunized with COL5A2 (Figure S5) or KRT 9 (Figure S6).

### Discussion

The cornerstone of adaptive immune responses is antigen-specificity. Extending our previous report (15), we investigated the potential influence of sex on the CAD patient immune peptidome by capturing soluble HLA Class-I/peptide complexes in female and male patients over the age of 50 years. Our results show that the patient immune peptidome have commonality between sexes, but also suggest that there may be a degree of uniqueness influenced by sex. It is important to note that although the self-peptides were identified using immune-precipitation of HLA Class-I, it does not restrict the immune response to the self-peptides to CD8+ T cells only. CD4+ T helper cells interact with CD8+ T cells in response to specific antigens to generate effector and memory responses (26), mediated by clustering interactions with a population of dendritic cells (27, 28) which optimizes the recall response by memory CD8+ T cells (29).

Differential manifestation of CAD between the sexes is recognized (3) yet specific mechanistic insights remain to be clarified. Inflammation is implicated in CAD (1) with relatively limited understanding of the processes involved. The chronic nature of CAD would lead to the reasonable speculation that the inflammatory response would evolve to implicate the adaptive immune response. Indeed, clinical studies not only identify T cells potentially involved in the disease (2) but also localize these T cells in various stages of the disease (30, 31). Interestingly, sexual dimorphism is also reported in immune responses (4, 5, 14).

The results of the peptide screening in apoE–/– mice (15) were intriguing in that there appeared to be differential immune responses to the peptide stimulation between the sexes. This differential outcome influenced by sex did not extend to aortic atherosclerosis in the context of our experimental paradigm of feeding the mice with high fat diet for 12 weeks since aortic plaque burden was similar between the sexes. Aortic sinus plaque size in females, however, were larger consistent with previous reports that suggest this is site-specific and may not reflect total disease burden (32, 33).

In agreement with our observation, high fat diet has been reported to induce a Type-2 cytokine response in male apoE–/– mice (34) but the response has not been investigated in the context of host sex. Our results also showed that although Pdcd1 and Ctla4 mRNA expression differed between the sexes, the extent of aortic atherosclerotic burden was similar. Given the differential immune profile between the sexes in response to self-peptides observed in the screening experiment, we challenged the self-reactive immune response to the self-peptides using immunization.

We identified self-peptides from patients that were common to both females and males and peptides that were unique to each sex, yet neither of the sex-unique peptides had significant effects on atherosclerosis in immunized mice of the corresponding sex. The implications of patient-unique peptides that were detected in only one sex remains unclear. However, sex-differential expression and function of proteins have been previously reported. Estrogen and androgens differentially affect transcription of collagen types I, III and V in mice (35). SAA3 deletion in mice revealed sexually dimorphic effects on inflammatory responses (36). Female rheumatoid arthritis patients with cardiovascular disease had significantly lower SAA levels compared to male patients (37). Expression levels of self-antigens as well as expression patterns of self-peptides are determinants of whether tolerance is established or self-reactivity is provoked (38, 39). Thus, protein amount, function, and tissue localization, any of which can be influenced by the sex of the host (35–37), may affect immune reactivity to self-antigens (38, 39). In addition, sex-bias occurs in class-I HLA mediated responses (14).

On the other hand, immunization with the peptide common to majority of female and male patients, COL6A1, had divergent effects on atherosclerosis. The differential outcome unmasked by COL6A1 immunization is further demonstrated by different immune profiles between the sexes. The reduction in atherosclerosis in immunized female mice is associated with increased CD8+IFN-γ+ T cells and reduced cytolytic activity. This profile of CD8+ T cells in immunized female mice remains to be investigated further. However, there are CD8+ T cell sub-types that are detrimental and there are others that promote favorable effects against atherosclerosis (40). Pro-atherogenic effects of CD8+ T cells are attributed in part to cytolytic activity which leads to increased expression of IL-1β (41, 42), resulting in an amplification loop promoting increased T cell activation by IL-1β (43). IL-1β is a proinflammatory cytokine mediator of atherosclerosis (44). COL6A1 immunization in female mice interrupted this pathway with a tolerance-like response of decreased CD8+ T cell cytolytic activity and reduced IL-1β expression with decreased aortic atherosclerosis (43).

Increased CD4+ T cell function is associated with worsening of experimental atherosclerosis (2), which we observed in the male COL6A1-immunized mice. Effector T cell function that exacerbates atherosclerosis is attributed in part to IFN-γ (45), which concur with our observation that COL6A1 immunized male mice had increased CD4+ EM T cells and CD4+IFN-γ+ T cells. Effector function by cognate T cell receptor-reactivated memory CD4+ T cells requires IL-1β signaling, potentiating the detrimental role of activated effector memory CD4+ T cells (46), in agreement with our results. IL-1β signaling was reported to promote a Th17 response in CD4+ T cells in atherosclerosis (47). This did not occur in our study likely due to increased Ctla4 mRNA expression that suppresses Th17 differentiation (48) and may be a compensatory response to the unfavorable effect of immunization in male mice. The lack of difference in serum IL-1β in immunized male mice despite increased mRNA expression could be due to several factors including increased consumption by activated CD4+EM T cells (46, 47) in COL6A1 immunized male mice.

It is notable that the immunologic challenge was necessary to promote differences in aortic plaque atherosclerosis between the sexes. The divergent response to COL6A1 immunization between the sexes may be linked to the preponderance of increased regulatory immune checkpoint mRNA expression observed in naive female mice fed high fat diet for 12 weeks. Challenge with a self-antigen unmasked the potential role of these differences. These remain speculative and need to be tested but underscore context-dependent antigen-specificity of immune responses in atherosclerosis.

The lack of seroconversion in COL6A1 immunized mice is not unexpected given that the immune challenge is directed to a self-antigen. Three components necessary to induce seroconversion to self-peptide immunization are: (1) A TLR agonist; (2) The use of an adjuvant as antigen depot; and (3) A short foreign peptide sequence (49). The immunization formulation used in our study, selected based on the report that it enhances CD8+ T cell responses (50), contained only the first 2 components which induced T cell responses to COL6A1 but no seroconversion.

COL6A1 is a member of the collagen superfamily that forms a triple-helix monomer with COL6A2 and COL6A3 and then assembles as tetramers secreted into the extracellular space (51). It is present in advanced lesions (52) and identified as a specific autoantigen in atherosclerosis associated with experimental autoimmunity (53), supporting the notion that chronic inflammatory conditions lead to the generation of self-reactive immune responses to COL6A1. Importantly, the report suggests that exaggerated immune challenges in female mice can result in pathologic responses to COL6A1 associated with the severity of atherosclerosis (53). Our results complement their report. It remains unclear how COL6A1 becomes a target of self-reactive immune responses in atherosclerosis. A report on proteomic analysis of atherosclerosis progression in apoE–/– mice may provide some insight. Although COL6A1 protein levels did not change as aortic plaques developed over time, it was the most abundant protein in the aorta (54). In addition, increased COL6A1 was found in the proteome of atherosclerotic coronary arteries compared to pre-atherosclerotic radial arteries (55). These reports suggest that COL6A1 is a source of potential atherosclerosis antigens during a chronic inflammatory state. It is noteworthy that decellularized equine arteries, under investigation as potential biocompatible scaffolding for engineered vascular tissue, retain immunogenicity attributable to COL6 (56).

It is known that clinical atherosclerosis is different in its prevalence, clinical presentation, and outcome between male and female patients (57) traditionally explained by the difference in plaque morphology, prevalence of risk factors, hormonal response, or lipids in epidemiology and clinical studies or preclinical animal models. Studies of potential sexual dimorphism in self-antigens, or immune responses to self-antigens in atherosclerosis are scant. Our results provide some evidence to fill in the gap.

### Limitations of the Study

The small size of plasma samples may be perceived as a limitation. However, the immune-peptidomic profiling is not intended to define biomarkers, nor is it intended to develop best-fit models for population-based stratification or prognostication, typical of large-scale proteomic studies of patient populations. Instead, it is intended to be used as a tool to identify potential self-reactive peptides that may help define specific pathways of inflammatory signaling mediated through the adaptive immune response (15) which can be tested for functionality in a mouse model. A limitation is the propensity of the control group to be younger, unavoidably inherent in the samples from self-reported control blood donors, some of whom cannot be ruled out as potentially undiagnosed CAD. It is important to note that the plasma samples were selected from patients over the age of 50 years, which presumably would place the females of the group in menopausal or post-menopausal status. In comparison, the female mice used in the study are in their reproductive age. The potential role of hormone status remains to be investigated further. The method to immune-precipitate MHC-I/peptide complexes is unlikely to pull down and identify peptides of small quantities that may nevertheless be important. Sex-influenced differences in immunologic profiles of mice in our report may be limited to the strain of mice used and inferences should be drawn carefully. The lack of observable difference in sex-unique peptide immunization may reflect a limitation of the mouse model. The pathogenesis of the disease may not be translatable for all potential pathways between mouse models and humans.

## Data Availability Statement

The datasets for this study are included in the article/Supplementary Material.

## Ethics Statement

The studies involving human participants were reviewed and approved by IRB-Cedars-Sinai Medical Center. Written informed consent for participation was not required for this study in accordance with the national legislation and the institutional requirements. The animal study was reviewed and approved by Institutional Animal Care and Use Committee, Cedars-Sinai Medical Center.

## Author Contributions

WL, BC, and PD contributed conception and design of the study. WL, JY, WY, JG, XZ, JZ, BZ, and PD contributed to data acquisition and analysis. WL, BC, WY, MF, K-YC, PS, and PD contributed to interpretation of data, drafting, and revising the manuscript.

## Conflict of Interest

The authors declare that the research was conducted in the absence of any commercial or financial relationships that could be construed as a potential conflict of interest.

## References

[1] Ridker PM Everett BM Thuren T MacFadyen JG Chang WH Ballantyne C. Antiinflammatory therapy with canakinumab for atherosclerotic disease. N Engl J Med. (2017) 377:1119–31. 10.1056/NEJMoa1707914 28845751

[2] Hansson GK Jonasson L. The discovery of cellular immunity in the atherosclerotic plaque. Arterioscler Thromb Vasc Biol. (2009) 29:1714–17. 10.1161/ATVBAHA.108.179713 19846836

[3] Bairey Merz CN Shaw LJ Reis SE Bittner V Kelsey SF Olson M. Insights from the NHLBI-Sponsored women's ischemia syndrome evaluation (WISE) study: part II: gender differences in presentation, diagnosis, and outcome with regard to gender-based pathophysiology of atherosclerosis and macrovascular and microvascular coronary disease. J Am Coll Cardiol. (2006) 47(Suppl. 3):S21–9. 10.1016/j.jacc.2004.12.084 16458167

[4] Lamason R Zhao P Rawat R Davis A Hall JC Chae JJ. Sexual dimorphism in immune response genes as a function of puberty. BMC Immunol. (2006) 7:2. 10.1186/1471-2172-7-2 16504066 PMC1402325

[5] Rubtsova K Marrack P Rubtsov AV. Sexual dimorphism in autoimmunity. J Clin Invest. (2015) 125:2187–93. 10.1172/JCI78082 25915581 PMC4497744

[6] Klein SL Marriott I Fish EN. Sex-based differences in immune function and responses to vaccination. Trans R Soc Trop Med Hyg. (2015) 109:9–15. 10.1093/trstmh/tru167 25573105 PMC4447843

[7] Bjorkbacka H Lavant EH Fredrikson GN Melander O Berglund G Carlson JA. Weak associations between human leucocyte antigen genotype and acute myocardial infarction. J Intern Med. (2010) 268:50–8. 10.1111/j.1365-2796.2009.02209.x 20141563

[8] Davies RW Wells GA Stewart AF Erdmann J Shah SH Ferguson JF. A genome-wide association study for coronary artery disease identifies a novel susceptibility locus in the major histocompatibility complex. Circ Cardiovasc Genet. (2012) 5:217–25. 10.1161/CIRCGENETICS.111.961243 22319020 PMC3335297

[9] Finn OJ. Human tumor antigens yesterday, today, and tomorrow. Cancer Immunol Res. (2017) 5:347–54. 10.1158/2326-6066.CIR-17-0112 28465452 PMC5490447

[10] Fredrikson GN Soderberg I Lindholm M Dimayuga P Chyu KY Shah PK. Inhibition of atherosclerosis in apoE-null mice by immunization with apoB-100 peptide sequences. Arterioscler Thromb Vasc Biol. (2003) 23:879–84. 10.1161/01.ATV.0000067937.93716.DB 12649092

[11] Mihailovic PM Lio WM Yano J Zhao X Zhou J Chyu KY. The cathelicidin protein CRAMP is a potential atherosclerosis self-antigen in ApoE(-/-) mice. PLoS ONE. (2017) 12:e0187432. 10.1371/journal.pone.0187432 29091929 PMC5665601

[12] Kimura T Kobiyama K Winkels H Tse K Miller J Vassallo M. Regulatory CD4(+) T cells recognize major histocompatibility complex class ii molecule-restricted peptide epitopes of apolipoprotein B. Circulation. (2018) 138:1130–43. 10.1161/CIRCULATIONAHA.117.031420 29588316 PMC6160361

[13] Dhanji S Chow MT Teh HS. Self-antigen maintains the innate antibacterial function of self-specific CD8 T cells *in vivo*. J Immunol. (2006) 177:138–46. 10.4049/jimmunol.177.1.138 16785508

[14] Schneider-Hohendorf T Gorlich D Savola P Kelkka T Mustjoki S Gross CC. Sex bias in MHC I-associated shaping of the adaptive immune system. Proc Natl Acad Sci USA. (2018) 115:2168–73. 10.1073/pnas.1716146115 29440397 PMC5834686

[15] Mihailovic PM Lio WM Herscovici R Chyu KY Yano J Zhao X. Keratin 8 is a potential self-antigen in the coronary artery disease immunopeptidome: a translational approach. PLoS ONE. (2019) 14:e0213025. 10.1371/journal.pone.0213025 30811493 PMC6392305

[16] Cercek B Shah PK Noc M Zahger D Zeymer U Matetzky S. Effect of short-term treatment with azithromycin on recurrent ischaemic events in patients with acute coronary syndrome in the azithromycin in acute coronary syndrome (AZACS) trial: a randomised controlled trial. Lancet. (2003) 361:809–813. 10.1016/S0140-6736(03)12706-7 12642046

[17] Morley S You S Pollan S Choi J Zhou B Hager MH. Regulation of microtubule dynamics by DIAPH3 influences amoeboid tumor cell mechanics and sensitivity to taxanes. Sci Rep. (2015) 5:12136. 10.1038/srep12136 26179371 PMC4503992

[18] Olsen JV de Godoy LM Li G Macek B Mortensen P Pesch R. Parts per million mass accuracy on an orbitrap mass spectrometer via lock mass injection into a C-trap. Mol Cell Proteomics. (2005) 4:2010–21. 10.1074/mcp.T500030-MCP200 16249172

[19] Cox J Neuhauser N Michalski A Scheltema RA Olsen JV Mann M. Andromeda: a peptide search engine integrated into the maxquant environment. J Proteome Res. (2011) 10:1794–805. 10.1021/pr101065j 21254760

[20] Cox J Mann M. MaxQuant enables high peptide identification rates, individualized p.p.b.-range mass accuracies and proteome-wide protein quantification. Nat Biotechnol. (2008) 26:1367–72. 10.1038/nbt.1511 19029910

[21] Kim Y Ponomarenko J Zhu Z Tamang D Wang P Greenbaum J. Immune epitope database analysis resource. Nucleic Acids Res. (2012) 40:W525–30. 10.1093/nar/gks438 22610854 PMC3394288

[22] Dimayuga PC Zhao X Yano J Lio WM Zhou J Mihailovic PM. Identification of apoB-100 peptide-specific CD8+ T cells in atherosclerosis. J Am Heart Assoc. (2017) 6:e005318. 10.1161/JAHA.116.005318 28711866 PMC5586274

[23] Hassan C Chabrol E Jahn L Kester MG de Ru AH Drijfhout JW. Naturally processed non-canonical HLA-A^*^02:01 presented peptides. J Biol Chem. (2015) 290:2593–603. 10.1074/jbc.M114.607028 25505266 PMC4317018

[24] Burrows SR Rossjohn J McCluskey J. Have we cut ourselves too short in mapping CTL epitopes? Trends Immunol. (2006) 27:11–6. 10.1016/j.it.2005.11.001 16297661

[25] Wu MS Li CH Ruppert JG Chang CC. Cytokeratin 8-MHC class I interactions: a potential novel immune escape phenotype by a lymph node metastatic carcinoma cell line. Biochem Biophys Res Commun. (2013) 441:618–23. 10.1016/j.bbrc.2013.10.105 24183726

[26] Castellino F Germain RN. Cooperation between CD4+ and CD8+ T cells: when, where, and how. Annu Rev Immunol. (2006) 24:519–40. 10.1146/annurev.immunol.23.021704.115825 16551258

[27] Eickhoff S Brewitz A Gerner MY Klauschen F Komander K Hemmi H. Robust anti-viral immunity requires multiple distinct T cell-dendritic cell interactions. Cell. (2015) 162:1322–37. 10.1016/j.cell.2015.08.004 26296422 PMC4567961

[28] Hor JL Whitney PG Zaid A Brooks AG Heath WR Mueller SN. Spatiotemporally distinct interactions with dendritic cell subsets facilitates CD4+ and CD8+ T cell activation to localized viral infection. Immunity. (2015) 43:554–65. 10.1016/j.immuni.2015.07.020 26297566

[29] Ahrends T Busselaar J Severson TM Babala N de VE Bovens A. CD4(+) T cell help creates memory CD8(+) T cells with innate and help-independent recall capacities. Nat Commun. (2019) 10:5531–13438. 10.1038/s41467-019-13438-1 31797935 PMC6892909

[30] Ammirati E Cianflone D Vecchio V Banfi M Vermi AC De MM. Effector memory T cells are associated with atherosclerosis in humans and animal models. J Am Heart Assoc. (2012) 1:27–41. 10.1161/xJAHA.111.000125 23130116 PMC3487313

[31] van Dijk RA Duinisveld AJ Schaapherder AF Mulder-Stapel A Hamming JF Kuiper J. A change in inflammatory footprint precedes plaque instability: a systematic evaluation of cellular aspects of the adaptive immune response in human atherosclerosis. J Am Heart Assoc. (2015) 4:e001403. 10.1161/JAHA.114.001403 25814626 PMC4579929

[32] Caligiuri G Nicoletti A Zhou X Tornberg I Hansson GK. Effects of sex and age on atherosclerosis and autoimmunity in apoE-deficient mice. Atherosclerosis. (1999) 145:301–8. 10.1016/S0021-9150(99)00081-7 10488957

[33] Teupser D Pavlides S Tan M Gutierrez-Ramos JC Kolbeck R Breslow JL Major reduction of atherosclerosis in fractalkine (CX3CL1)-deficient mice is at the brachiocephalic artery, not the aortic root. Proc Natl Acad Sci USA. (2004) 101:17795–800. 10.1073/pnas.0408096101 15596719 PMC539720

[34] Zhou X Paulsson G Stemme S Hansson GK. Hypercholesterolemia is associated with a T helper (Th) 1/Th2 switch of the autoimmune response in atherosclerotic apo E-knockout mice. J Clin Invest. (1998) 101:1717–25. 10.1172/JCI1216 9541503 PMC508754

[35] Markiewicz M Asano Y Znoyko S Gong Y Watson DK Trojanowska M. Distinct effects of gonadectomy in male and female mice on collagen fibrillogenesis in the skin. J Dermatol Sci. (2007) 47:217–26. 10.1016/j.jdermsci.2007.05.008 17601707 PMC2717737

[36] den Hartigh LJ Wang S Goodspeed L Ding Y Averill M Subramanian S. Deletion of serum amyloid A3 improves high fat high sucrose diet-induced adipose tissue inflammation and hyperlipidemia in female mice. PLoS ONE. (2014) 9:e108564. 10.1371/journal.pone.0108564 25251243 PMC4177399

[37] Targonska-Stepniak B Majdan M. Serum amyloid a as a marker of persistent inflammation and an indicator of cardiovascular and renal involvement in patients with rheumatoid arthritis. Mediators Inflamm. (2014) 2014:793628. 10.1155/2014/793628 25525305 PMC4265690

[38] Swee LK Nusser A Curti M Kreuzaler M Rolink H Terracciano L. The amount of self-antigen determines the effector function of murine T cells escaping negative selection. Eur J Immunol. (2014) 44:1299–312. 10.1002/eji.201343840 24497074

[39] Malhotra D Linehan JL Dileepan T Lee YJ Purtha WE Lu JV. Tolerance is established in polyclonal CD4(+) T cells by distinct mechanisms, according to self-peptide expression patterns. Nat Immunol. (2016) 17:187–95. 10.1038/ni.3327 26726812 PMC4718891

[40] van DJ Kuiper J Slutter B The many faces of CD8+ T cells in atherosclerosis. Curr Opin Lipidol. (2018) 29:411–6. 10.1097/MOL.0000000000000541 30020198

[41] Kyaw T Winship A Tay C Kanellakis P Hosseini H Cao A. Cytotoxic and proinflammatory CD8+ T lymphocytes promote development of vulnerable atherosclerotic plaques in apoE-deficient mice. Circulation. (2013) 127:1028–39. 10.1161/CIRCULATIONAHA.112.001347 23395974

[42] Novais FO Carvalho AM Clark ML Carvalho LP Beiting DP Brodsky IE. CD8+ T cell cytotoxicity mediates pathology in the skin by inflammasome activation and IL-1beta production. PLoS Pathog. (2017) 13:e1006196. 10.1371/journal.ppat.1006196 28192528 PMC5325592

[43] Yao Y Chen S Cao M Fan X Yang T Huang Y. Antigen-specific CD8(+) T cell feedback activates NLRP3 inflammasome in antigen-presenting cells through perforin. Nat Commun. (2017) 8:15402. 10.1038/ncomms15402 28537251 PMC5458103

[44] Kirii H Niwa T Yamada Y Wada H Saito K Iwakura Y. Lack of interleukin-1beta decreases the severity of atherosclerosis in ApoE-deficient mice. Arterioscler Thromb Vasc Biol. (2003) 23:656–60. 10.1161/01.ATV.0000064374.15232.C3 12615675

[45] Zhou X Nicoletti A Elhage R Hansson GK. Transfer of CD4(+) T cells aggravates atherosclerosis in immunodeficient apolipoprotein E knockout mice. Circulation. (2000) 102:2919–22. 10.1161/01.CIR.102.24.2919 11113040

[46] Jain A Song R Wakeland EK Pasare C. T cell-intrinsic IL-1R signaling licenses effector cytokine production by memory CD4 T cells. Nat Commun. (2018) 9:3185. 10.1038/s41467-018-05489-7 30093707 PMC6085393

[47] Engelbertsen D Rattik S Wigren M Vallejo J Marinkovic G Schiopu A. IL-1R and MyD88 signalling in CD4+ T cells promote Th17 immunity and atherosclerosis. Cardiovasc Res. (2018) 114:180–7. 10.1093/cvr/cvx196 29036304

[48] Ying H Yang L Qiao G Li Z Zhang L Yin F. Cutting edge: CTLA-4–B7 interaction suppresses Th17 cell differentiation. J Immunol. (2010) 185:1375–8. 10.4049/jimmunol.0903369 20601598 PMC2915549

[49] Saupe F Huijbers EJ Hein T Femel J Cedervall J Olsson AK. Vaccines targeting self-antigens: mechanisms and efficacy-determining parameters. FASEB J. (2015) 29:3253–62. 10.1096/fj.15-271502 25868727

[50] MacLeod MK McKee AS David A Wang J Mason R Kappler JW. Vaccine adjuvants aluminum and monophosphoryl lipid a provide distinct signals to generate protective cytotoxic memory CD8 T cells. Proc Natl Acad Sci USA. (2011) 108:7914–9. 10.1073/pnas.1104588108 21518876 PMC3093483

[51] Cescon M Gattazzo F Chen P Bonaldo P. Collagen VI at a glance. J Cell Sci. (2015) 128:3525–31. 10.1242/jcs.169748 26377767

[52] Katsuda S Okada Y Minamoto T Oda Y Matsui Y Nakanishi I. Collagens in human atherosclerosis. Immunohistochemical analysis using collagen type-specific antibodies. Arterioscler Thromb. (1992) 12:494–502. 10.1161/01.ATV.12.4.494 1373075

[53] Merched AJ Daret D Li L Franzl N Sauvage-Merched M. Specific autoantigens in experimental autoimmunity-associated atherosclerosis. FASEB J. (2016) 30:2123–34. 10.1096/fj.201500131 26891734

[54] Wierer M Prestel M Schiller HB Yan G Schaab C Azghandi S. Compartment-resolved proteomic analysis of mouse aorta during atherosclerotic plaque formation reveals osteoclast-specific protein expression. Mol Cell Proteomics. (2018) 17:321–34. 10.1074/mcp.RA117.000315 29208753 PMC5795394

[55] de la CF varez-Llamas G Maroto AS Donado A Zubiri I Posada M A proteomic focus on the alterations occurring at the human atherosclerotic coronary intima. Mol Cell Proteomics. (2011) 10:M110 10.1074/mcp.M110.003517 PMC3069343 21248247

[56] Boeer U Buettner FF Klingenberg M Antonopoulos GC Meyer H Haverich A. Immunogenicity of intensively decellularized equine carotid arteries is conferred by the extracellular matrix protein collagen type VI. PLoS ONE. (2014) 9:e105964. 10.1371/journal.pone.0105964 25157402 PMC4144968

[57] Robinet P Milewicz DM Cassis LA Leeper NJ Lu HS Smith JD. Consideration of sex differences in design and reporting of experimental arterial pathology studies-statement from ATVB council. Arterioscler Thromb Vasc Biol. (2018) 38:292–303. 10.1161/ATVBAHA.117.309524 29301789 PMC5785439

