# Supplementary material for: Sex as a Determinant of Responses to a Coronary Artery Disease Self-Antigen Identified by Immune-Peptidomics
Source: Front Immunol. 2020 Apr 21;11:694. doi: 10.3389/fimmu.2020.00694 (PMC7187896; doi:10.3389/fimmu.2020.00694)
Supplement: Supplementary file 1 [file Table_1.pdf]

Supplemental Table I: Peptides unique to Patients					
**Peptide sequence**	**Gene name**	**Male**	**Female**	**combined**	**Frequency (%)**
PGDDNNDIAPR	COL6A1	8	5	13	65
SSGGGSSGSSSGSSIAQGGSAGS	CDSN	4	1	5	25
SLADQAANEWGRSGKD	SAA1	4	1	5	25
SLADQAANKWGRSGRD	SAA2	4	1	5	25
ARENIQRFFGHGAED	SAA1	2	1	3	15
FRPAGLPEKY	SAA1;SAA2	3	0	3	15
MREANYIGSD	SAA1;SAA2-SAA4;SAA2	3	0	3	15
DGEAGAQGPPGPAGPA	COL1A1	0	2	2	10
GPSGPVGPPG	COL5A2	0	2	2	10
GSWQGVPGHNGAWETSGGHG	DMKN	2	0	2	10
KKNHKEEMSQLTGQNSGDVNVEIN	KRT9	2	0	2	10
LQYGGSANAESVQGVTPLHL	ANK1	1	0	1	5
QNKYQQAEELY	KLC3	1	0	1	5
RKQNDQRKVSQGR	MRVI1	1	0	1	5
			Both sexes		
			Female-unique		
			Male-unique		
