# Supplementary material for: Sex as a Determinant of Responses to a Coronary Artery Disease Self-Antigen Identified by Immune-Peptidomics
Source: Front Immunol. 2020 Apr 21;11:694. doi: 10.3389/fimmu.2020.00694 (PMC7187896; doi:10.3389/fimmu.2020.00694)
Supplement: Supplementary file 2 [file Table_2.pdf]

Supplemental Table II: Peptides unique to Controls	
**Sequence**	**Gene names**
NTYNRVSED	FGA
GHKEVTKEVVTSED	FGA
FESKSYKMA	FGA
PYKQGFGNVATNTDG	FGB
GSVDFGRKWD	FGB
VDFGRKWD	FGB
QQKQLEQVIAKD	FGA
EAAFFDTASTGK	FGA
PYKQGFGNVATNT	FGB
STFESKSYK	FGA
VTKEVVTSED	FGA
FSSANNRDNTYNRVSED	FGA
PSDKFFTSHNG	FGG
LCEQQTSD	DYM
EVTKEVVTSED	FGA
SVDFGRKWD	FGB
ARPAKAAATQKKVERKAPD	FGB
TQVNTQAEQLR	APOA4
PYKQGFGN	FGB
YAMFKVGPEAD	FGG
DAAYMNKV	KRT6B;KRT6C;KRT6A;KRT75;KRT72;KRT5
NGFKSHALQLNNR	C4B;C4A
DEPPQSPWD	APOA1
LGRQLTSGP	SERPINF2
SSYSKQFT	FGA
VREEPSND	C5orf42
STSYGTGSETESPRN	FGA
SYSKQFTSSTSYN	FGA
SPGSGNARPNNPDWG	FGA
PAPEVYAGR	HES4
YEDQQKQLE	FGA
PSILEMSR	APOL1
EITRGGSTSYGTG	FGA
SEGGFTATGQR	ECM1
NEEGFFSARGHRPLD	FGB
VNDNEEGFFSARGHRPLD	FGB
SPGSGNARPNN	FGA
QNPGSPRPGSTGTWN	FGA
DSTFESKSYKM	FGA
TASTGKTFPGF	FGA
SSKGSLGGGF	KRT10
VTKEVVTSEDG	FGA
EGGFTATGQR	ECM1
GWTVIQNRQD	FGB
FSTYDRDND	FGB
TVGSLAGQPLQER	APOE
EITRGGSTSYGTGSETESPRN	FGA
VDQHEWTK	TTN
NVIRDAVTY	HIST1H4A
ALTDMPQM	FGA
GDFNSYVR	C4B;C4A
SHSLTTNIME	FGA
VYLPTHTSL	MYLIP
