# Supplementary material for: Sex as a Determinant of Responses to a Coronary Artery Disease Self-Antigen Identified by Immune-Peptidomics
Source: Front Immunol. 2020 Apr 21;11:694. doi: 10.3389/fimmu.2020.00694 (PMC7187896; doi:10.3389/fimmu.2020.00694)
Supplement: Supplementary file 3 [file Table_3.pdf]

Supplemental Table III: Peptides common in at least one each control and patient	
**Sequence**	**Gene names**
STFESKSY	FGA
ASVSTVLTSKYR	HBA1
PYKQGFGNVATNTD	FGB
TLEIPGNSD	C4B;C4A
LASVSTVLTSKYR	HBA1
SVSTVLTSKYR	HBA1
TQLQEAQRELKEA	CEP250
HWESASLLR	C3
STVLTSKYR	HBA1
VSTVLTSKYR	HBA1
FLAEGGGVR	FGA
LDLSTKYR	IL17RD
GDSTFESKSY	FGA
HWESASLL	C3
YENEVALRQ	KRT10
NVKVDPEIQ	KRT2
SRQFSSRSGYRSGGGFSSGSAG	KRT1
SNTQAERSIIGM	S100A7A;S100A7
WVQKTIAEN	HP;HPR
AQYEDIAQK	KRT1
DTNADKQLSF	S100A9
GILNPSQPGQSSSSSQT	CDSN
SRSGGGGGGGLGSGGSIRSSY	KRT9
TLELGPGG	GRIN2C
DWAAGTMD	APOL1
DSTFESKSY	FGA
APRSALYSPSD	QSOX1
DFLAEGGGVR	FGA
DIVMTQSPD	IGKV4-1
DNTYNRVSED	FGA
AQYEDIAQKS	KRT1
EGDFLAEGGGVR	FGA
YQSKYEEL	KRT1
LDTNADKQLS	S100A9
SGGGGGGGLGSGGSIR	KRT9
KFLASVSTVLTSKYR	HBA1
NTDSPLRY	RPSA
DINTDGAVNF	S100A8
SRQFSSRSGY	KRT1
QEQLEQIR	RIBC2
EEAGARVQQNVPSGTDTGD	APOL1
SYSKQFTS	FGA
LQIDNARLA	KRT10;KRT19;KRT28;KRT18;KRT12;KRT17;KRT14
NVELDPEIQ	KRT71;KRT73;KRT74
KVPQVSTPTLVEVSR	ALB
YENEVALR	KRT10
GSGGGSYGSGGGGGGHGSY	KRT1
DSGEGDFLAEGGGVR	FGA
ASTSTTIRSH	KRT6B;KRT6C;KRT6A
SSSYSKQFTS	FGA
AQYEEIAQR	KRT2;KRT6B;KRT6C;KRT6A;KRT4
EEQLQQIRAE	KRT10
GGGGGGGLGSGGSIR	KRT9
ASTSTTIRSHSS	KRT6B;KRT6C;KRT6A
EEAGARVQQNVPSGTD	APOL1
SMDNNRSLDLD	KRT1;KRT77
VVTGNMGSNDKVGDF	DSG1
LDTNADKQL	S100A9
IINTFHQY	S100A9
DTAAQITQR	HLA-B
QKENAGEDPGLA	DCD
DKESVPISD	FN1
ALEEQLQQIR	KRT10
YEDIAQKS	KRT1
SLVNLGGSK	KRT1

ASVSTVLTSKY	HBA1
TGDPQSKPLGD	APOL1
EEANNDLENKIQ	KRT9
SLVNLGGSKSISIS	KRT1
EESNYELEGK	KRT10
KFIDTTSKF	RPL3;RPL3L
SYNRGDSTF	FGA
TPDVSSALD	APOC1
SSSKGSLGGGF	KRT10
SRQFSSRSGYRSGGGFSSGSA	KRT1
ASTSTTIRSHSSS	KRT6B;KRT6C;KRT6A
KPSQMQVTDVQD	FN1
SRQFSSRSGYRSGGGFSSGSAGIIN	KRT1
RNVSTGDVNVE	KRT10
AGSAFAVHD	HP;HPR
TEELAYLK	KRT10
SGGGGGGGLGSGGSIRSS	KRT9
GIGTVPVGRVE	EEF1A1P5;EEF1A1;EEF1A2
SRQFSSRSGYRSGGGFSSG	KRT1
AQNVGTTHDL	BLMH
NAENEFVTIK	KRT1
ALEEQLQQIRAE	KRT10
TEELNREVATN	KRT17;KRT14
DEVGGEALGRL	HBB
TNAENEFVTIK	KRT1
ASTIKENQFD	F5
YRSGGGFSSGSAGII	KRT1
YRSGGGFSSGSAGIIN	KRT1
NNMRSQYEQL	KRT10
FVKTGSSSGGD	ICE1
VDSGNDVTDIADD	HP
SYSKQFTSS	FGA
VSLQLPSR	C3
NALQSGNSQESVTEQD	IGKC
GEGDFLAEGGGVR	FGA
RSGGGFSSGSAGIIN	KRT1
SMDNSRNLDLD	KRT2
YRSGGGFSSGSAGI	KRT1
AQYEEIAQRS	KRT2;KRT6B;KRT6C;KRT6A;KRT4
SGEGDFLAEGGGVR	FGA
LNNMRSQYEQL	KRT10
SVPGPMGPSGPR	COL1A1
EGVQKEDIPPAD	C3
ALADGVQKV	APOL1
NSGDVNVEIN	KRT9
GDFLAEGGGVR	FGA
VSTGDVNVEMN	KRT10
DYGHTGYGPSGGS	FLG2
SRQFSSRSGYRSGGGFSSGS	KRT1
DTNADKQLSFEE	S100A9
AENEFVTLK	KRT6B;KRT6C;KRT6A
VVYPWTQR	HBB;HBD;HBG2;HBG1;HBE1
YQSKYEELQIT	KRT1
SSSKGSLGGGFSSG	KRT10
APEEHPVLL	ACTG1;ACTB;POTEF
SSSYSKQFT	FGA
SRQFSSRSGYRSGGGFSSGSAGI	KRT1
KESVPISD	FN1
AMGIMNSFVNDIFER	HIST1H2BL;HIST1H2BM;HIST1H2BN;HIST1H2BH;HIST3H2BB;HIST2H2BF;HIST2H2BE;HIST1H2BC;HIST1H2BD;<br>H2BFS;HIST1H2BB;HIST1H2BO;HIST1H2BJ;HIST1H2BK
GGGGGGGLGSGGSIRSS	KRT9
KPVDPDGPENGPP	DSC1
SSSKGSLGGGFSSGGFSGGS	KRT10
GIGTVPVGRVET	EEF1A1P5;EEF1A1;EEF1A2
YGAEALERMF	HBA1
SGGVFAVRD	C1R
RPPGFSPFR	KNG1

VPVTVTVHD	C3
ITQIEHEVSSS	KRT9
AAGPPISEGKY	CDSN
ASTSTTIRSHS	KRT6B;KRT6C;KRT6A
SYSKQFTSST	FGA
GSGSGWSSSRGPY	HRNR
FVSTTYSGVTR	KRT1
DNSRNLDLD	KRT2
KVEQAVETEPE	APOE
SRQFSSRSGYRSGGGFS	KRT1
RVQQNVPSGTDTGD	APOL1
AGELTPEEEAQ	CALML5
PRHRQGPVNLLSD	ITIH4
LTVGNNKTL	KRT9
SASSLGGGFGGGSRGFGGASGGGY	KRT9
TTEIDNNIE	KRT10
SRSGGGGGGGLGSGGSIRSSYSRF	KRT9
GGGGGGGLGSGGSIRSSY	KRT9
ALGNTGHEIG	DMKN
GVLNNPGPG	AZU1
LSRSGGGGGGGLGSGGSIRSSY	KRT9
KENAGEDPGLA	DCD
YDAEISQIHQ	KRT2
GIGTVPVGRVETG	EEF1A1P5;EEF1A1;EEF1A2
LEGEGSSGGGGRGGGSF	KRT10
GGGGGGLGSGGSIR	KRT9
RSGGGFSSGSAGI	KRT1
NVHSGSTFF	ITIH4
GGFGGGSFRGSYGSS	KRT10
ELIDQDARDL	ANXA2
SLGGGFGGGSRGFGGASGGGY	KRT9
LRVAPEEHPVL	ACTG1;ACTB;POTEF
RSGGGFSSGSAGII	KRT1
SSSKGSLGGGFSSGGFSGGSF	KRT10
GPVGPSGPPGKD	COL3A1
SSKGSLGGGFSSG	KRT10
DAGAGIALNDH	GAPDH
SYVRVTASD	C4B;C4A
SRQSSVSFR	KRT5
YRSGGGFSSGSAGIINY	KRT1
AELRAPPD	PCYOX1
PELTESSGSASHID	C9
LDSTVLNSHLLA	KIAA0232
KYSLIKGNF	S100A8
TPDVSSAL	APOC1
ASGNARIGKPAPD	PRDX2
NDMRQEYEQLIAK	KRT9
TSIDAHNGVAP	VRK1
SKAEAESLYQS	KRT1
SSSKGSLGGGFSSGGF	KRT10
PYTVSHYAVGD	FN1
FLASVSTVLTSKYR	HBA1
KYQEVTNNL	CAPRIN1
RNVSTGDVNVEMN	KRT10
HASDRIIAL	TKT
HPTDDTTTL	FXYD5
KDVDNAYMIK	KRT2
IREADIDGDGQVN	CALM1
SLVGLGGTK	KRT2
GGGGGGLGSGGSIRSS	KRT9
DSIIAEVKAQ	KRT1;KRT8;KRT2;KRT6B;KRT6C;KRT6A;KRT75;KRT79;KRT5
EIELQSQLALKQ	KRT10
SDKPDMAEIEKFDK	TMSB4X
TASNTFRVV	EVL
DIKIRLENE	KRT10
SKGKIYPVGY	CDSN
RSGGGGGGGLGSGGSIRSSY	KRT9

LVNLGGSKS	KRT1
VNLPINGNGKQ	GSTP1
KALDPEISSGEGL	DSC1
AGELTPEEEAQYKK	CALML5
GGGSFRGSYGSS	KRT10
LQIDNARL	KRT10;KRT19;KRT28;KRT18;KRT12;KRT25;KRT17;KRT14
RNVSTGDVNVEM	KRT10
LSRSGGGGGGGLGSGGSIRSSYSRF	KRT9
ALTNAVAHVD	HBA1
GYSFTTTAER	ACTG1;ACTB
SSSKGSLGGGFSSGG	KRT10
GGPGGFGPGGYPGGIHEV	KRT2
SEETKENEGF	C3
GSNDKVGDF	DSG1
DLDTNADKQLS	S100A9
MDCCTENACSK	NRGN
HPISDHEATL	HLA-B;HLA-H;HLA-F;HLA-E
NREVATNSEL	KRT17;KRT14
SSTISSNVASKA	KRT2
SSSYSKQFTSSTSYNRGD	FGA
NARLAADDF	KRT10;KRT19;KRT28;KRT18;KRT15;KRT13;KRT24;KRT17;KRT14
DINTDGAVN	S100A8
NEVLTKTY	NAP1L1
GGPGGFGPGGYPGGIH	KRT2
VNVEINVAPGKD	KRT9
SSSKGSLGGGFSS	KRT10
FEMEQNLRQ	KRT9
GSQGGLGGQGQGNPGGLGTP	DMKN
ISSSKGSLGGGF	KRT10
RSGGGFSSGSAGIINY	KRT1
SEILSDPSDDTKG	DSP
RGILNPSQPGQSSSSSQT	CDSN
KQFTSSTSY	FGA
ESSHGWTGPSTG	FLG
GGGGGSFGAGGGFGSRS	KRT1
RDYQELMNT	KRT1
SKELTTEIDNNIE	KRT10
YSGFGQHGSGSD	FLG2
SVKLGHPDTL	S100A9
LGHPDTLNQGE	S100A9
RQGVDADINGL	KRT9
SSGQYSGFGQHGSGSD	FLG2
YEEIAQRS	KRT2;KRT6B;KRT6C;KRT6A;KRT4
DEPPQSPWDRVKD	APOA1
RSALYSPSD	QSOX1
FPSIVGRPR	ACTG1;ACTB;POTEF;POTEE;POTEKP;ACTG2;ACTA1;ACTC1;ACTA2;POTEI
NLNDRLAS	KRT10;KRT19;KRT28;KRT12;KRT25;KRT15;KRT13;KRT23;KRT20;KRT27;KRT26;KRT17;KRT14
MNSLSEANTK	SERPINB4;SERPINB3
APTGDLPRA	PSMB9
SSHGWTGPSTG	FLG
EVVGGSSDSY	CDSN
KDIENQYETQ	KRT9
SPDGSDPKL	WDR48
TWKPGSSGP	FGA
AMGAATGVVTA	SLC9A6
FNSYVRVTASD	C4B;C4A
DSSEEKFLR	STATH
DGINSGITHAG	SBSN
QSKVDLLNQ	KRT2
DEHSVMTY	FLNA;FLNB;FLNC
AKLDNLQQ	KRT1
