# Supplementary figures and images for: Sex as a Determinant of Responses to a Coronary Artery Disease Self-Antigen Identified by Immune-Peptidomics
Source: Front Immunol. 2020 Apr 21;11:694. doi: 10.3389/fimmu.2020.00694 (PMC7187896; doi:10.3389/fimmu.2020.00694)

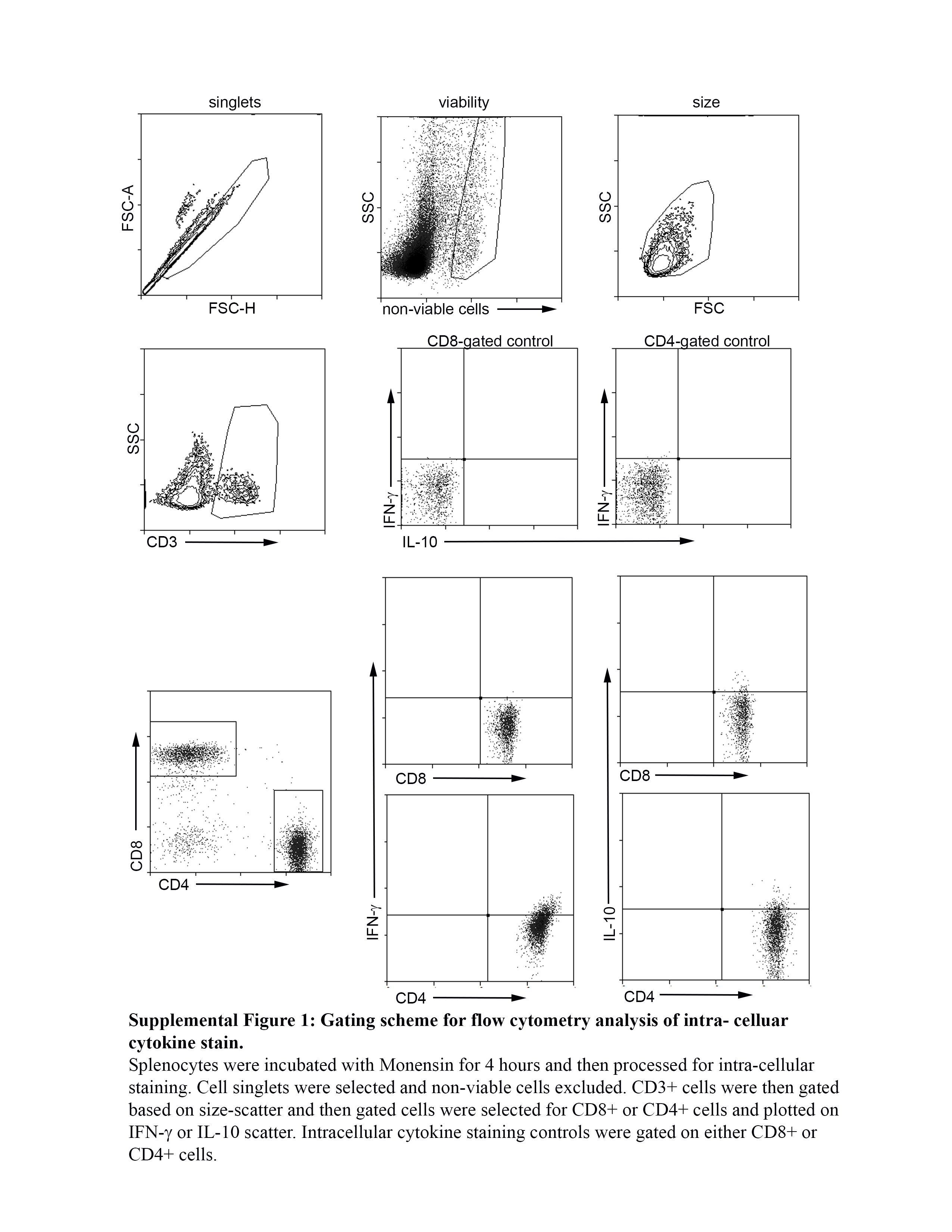

Supplement: Supplementary file 5 [file Image_1.TIF]

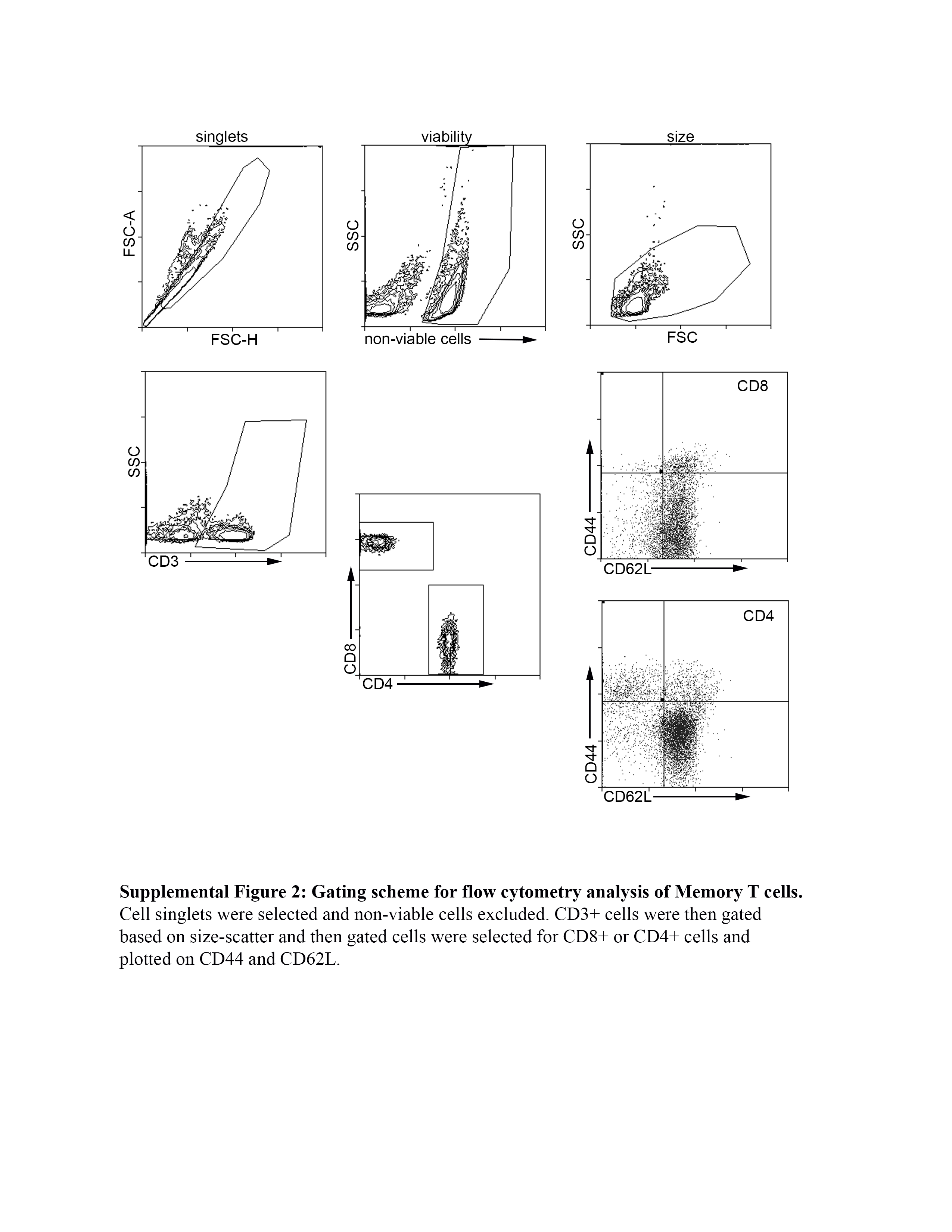

Supplement: Supplementary file 6 [file Image_2.TIF]

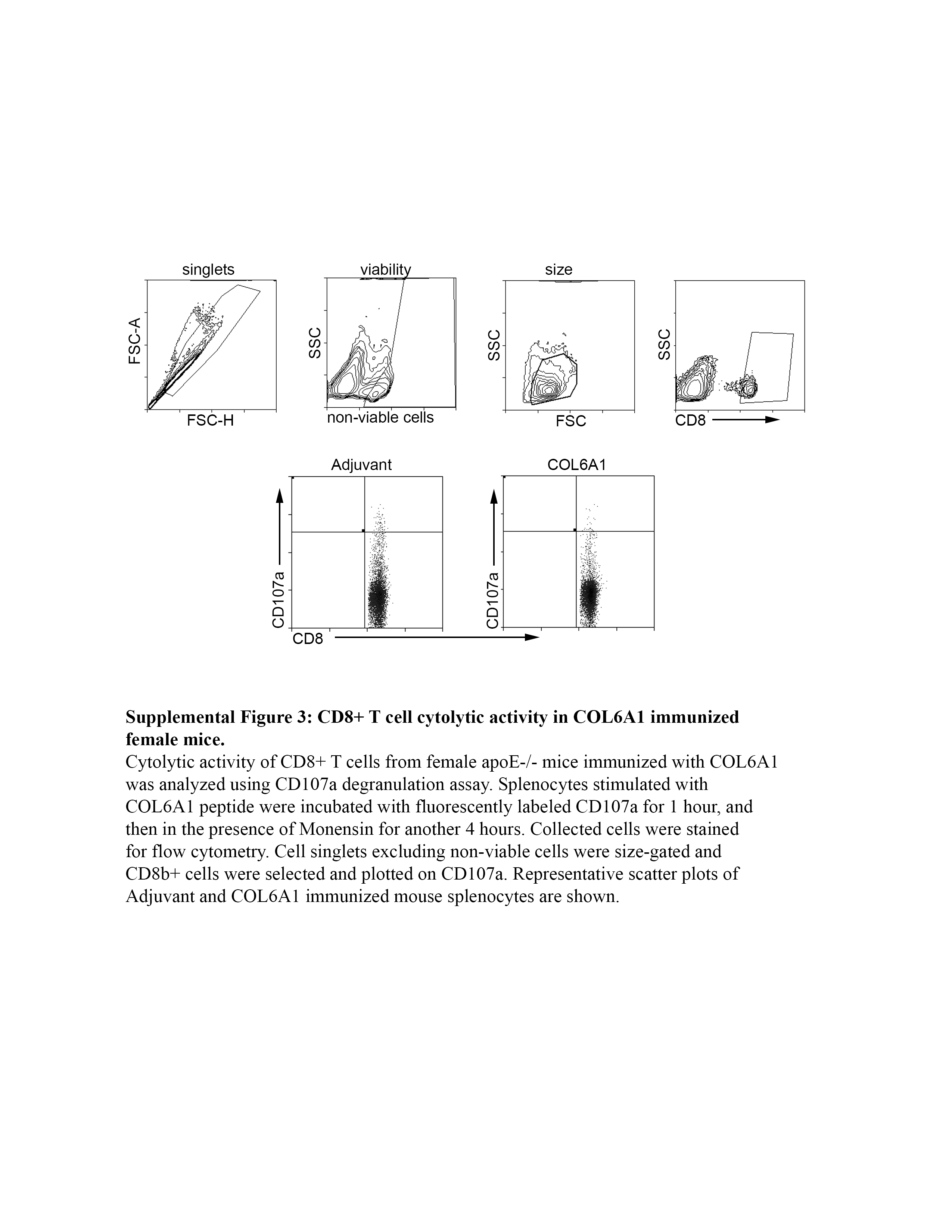

Supplement: Supplementary file 7 [file Image_3.TIF]

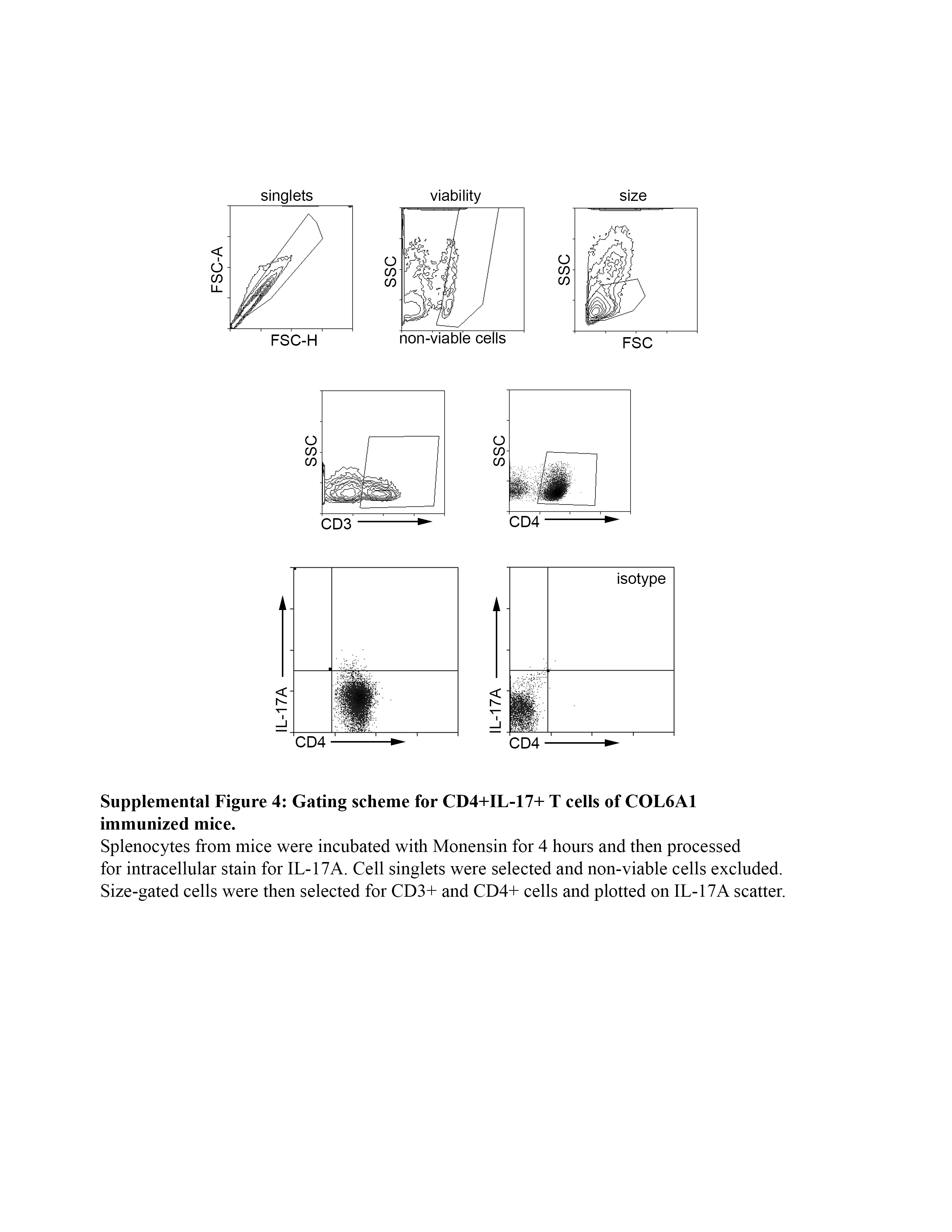

Supplement: Supplementary file 8 [file Image_4.TIF]

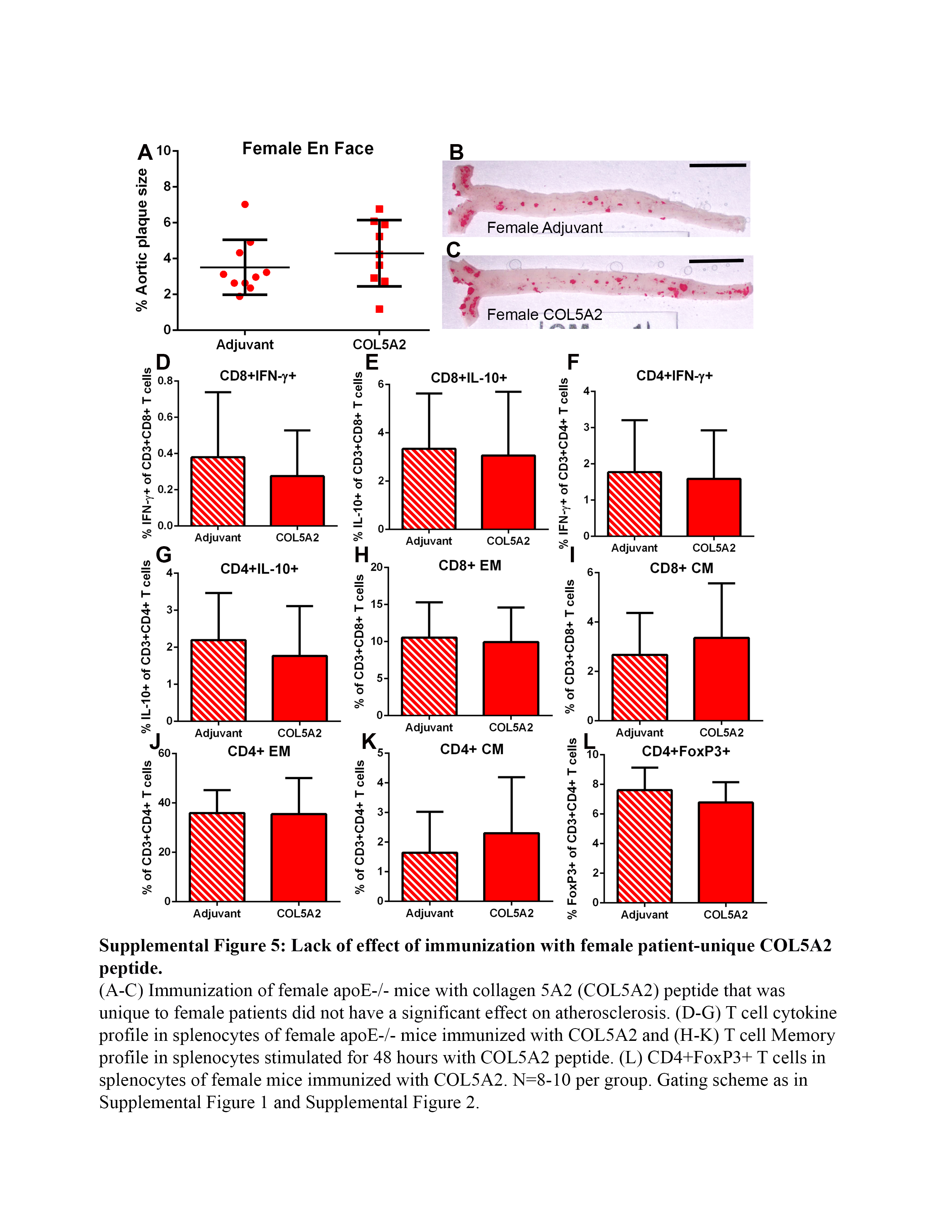

Supplement: Supplementary file 9 [file Image_5.TIF]

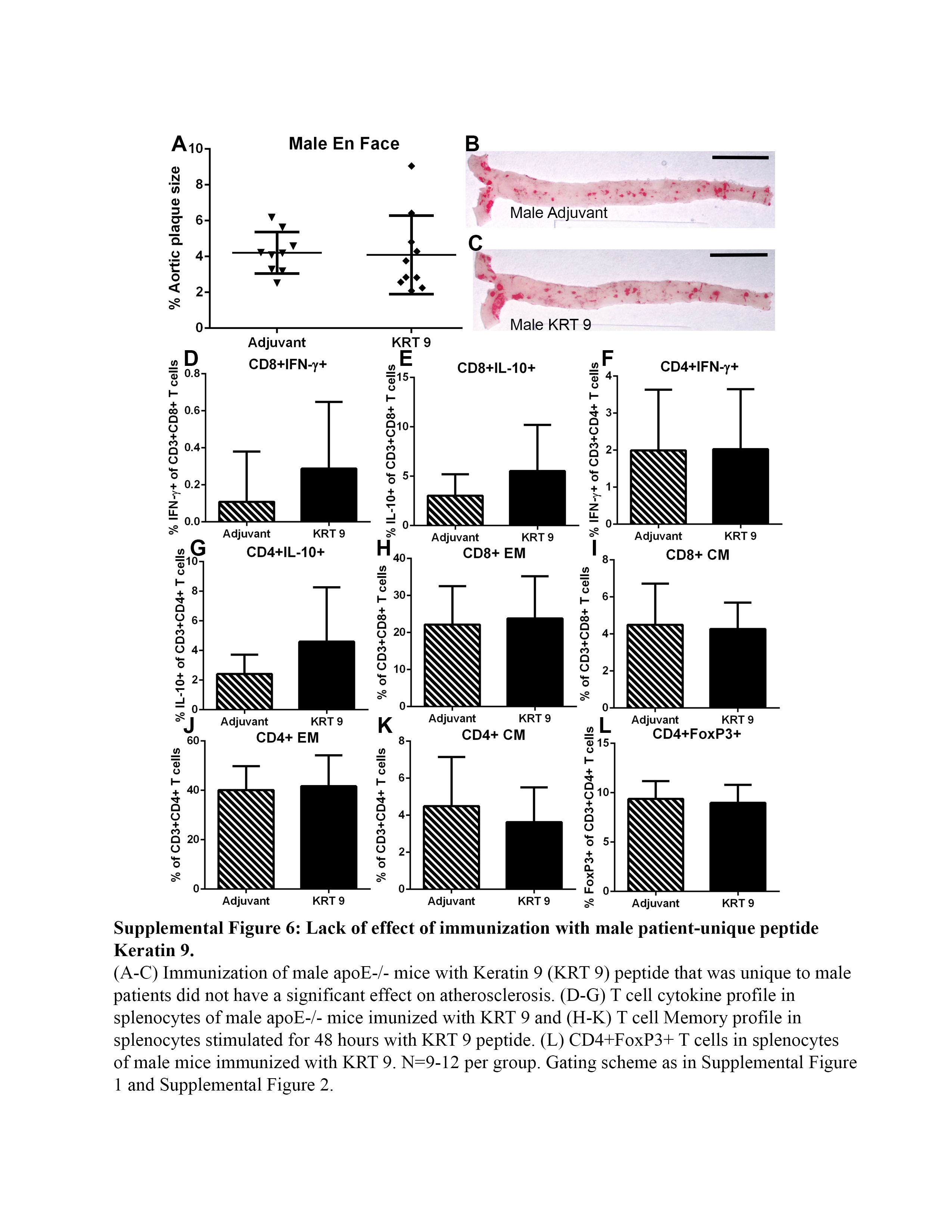

Supplement: Supplementary file 10 [file Image_6.TIF]
